# Supplementary material for: A Curriculum to Improve Pediatric Residents' Telephone Triage Skills
Source: MedEdPORTAL. 2020 Oct 22;16:10993. doi: 10.15766/mep_2374-8265.10993 (PMC7586755; doi:10.15766/mep_2374-8265.10993)
Supplement: Supplementary file 1 — Pediatric Phone Triage Conference Presentation.pptxFaculty Guide - Pediatric Phone Triage Conference.docxJust-in-Time Training.docxResident Cheat Sheet.docxPre- and Postexperience Self-Assessment.docxConvenience Sample Preassessment.docx [file mep_2374-8265.10993-s001.zip › A. Pediatric Phone Triage Conference Presentation.pptx]

## Slide 1
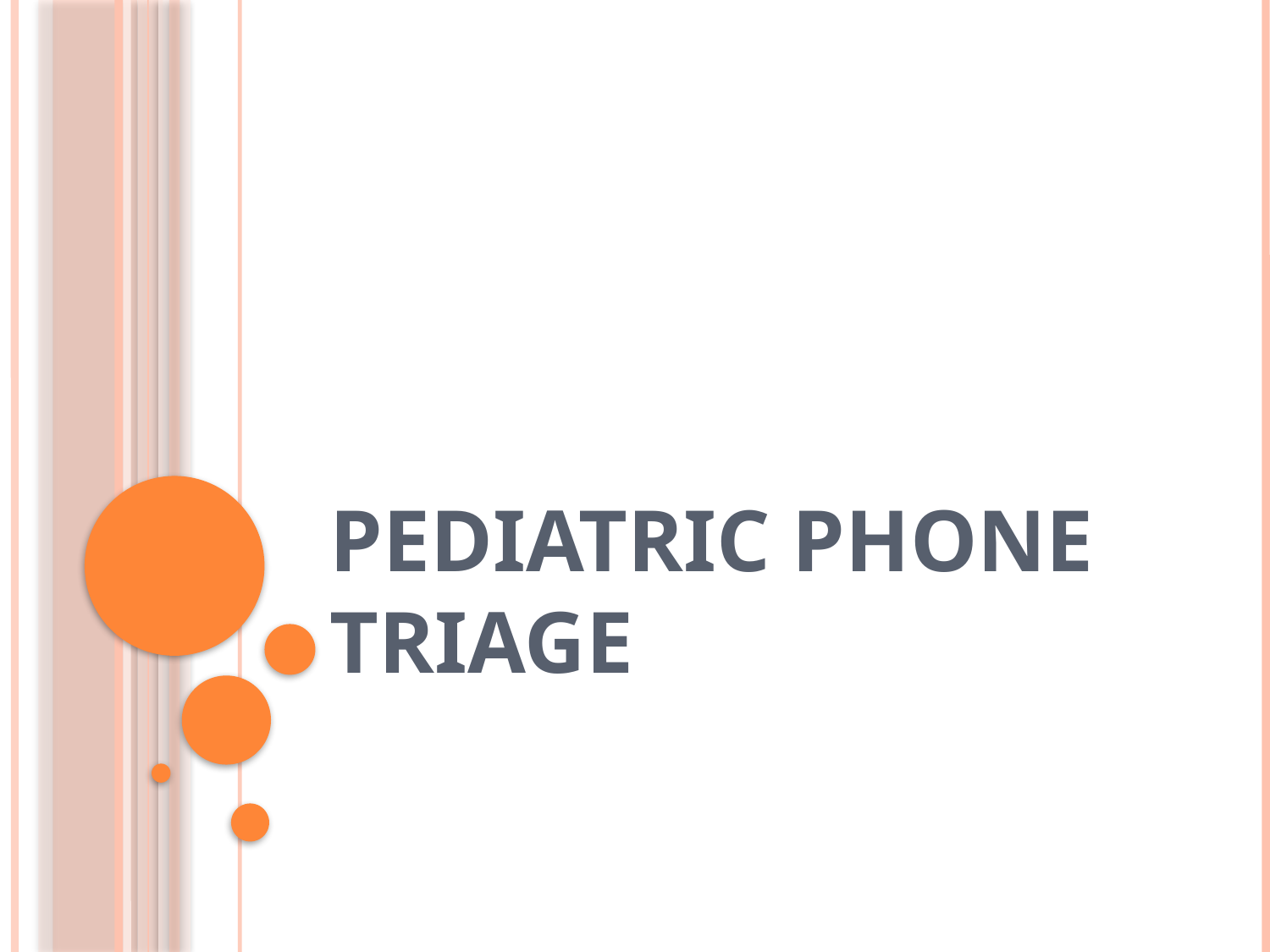

# Pediatric Phone Triage

## Slide 2
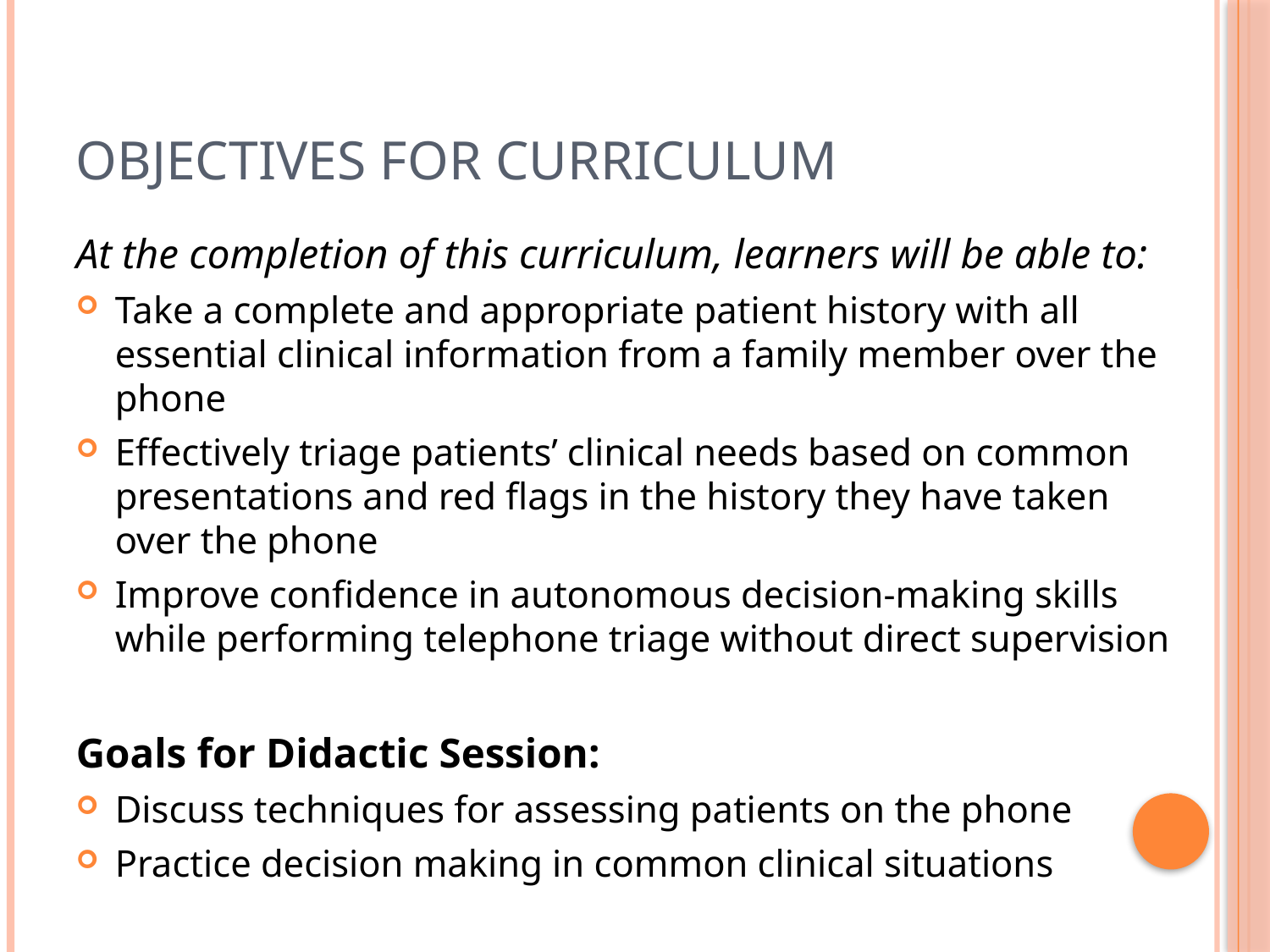

# Objectives For Curriculum
At the completion of this curriculum, learners will be able to:
Take a complete and appropriate patient history with all essential clinical information from a family member over the phone
Effectively triage patients’ clinical needs based on common presentations and red flags in the history they have taken over the phone
Improve confidence in autonomous decision-making skills while performing telephone triage without direct supervision
Goals for Didactic Session:
Discuss techniques for assessing patients on the phone
Practice decision making in common clinical situations

## Slide 3
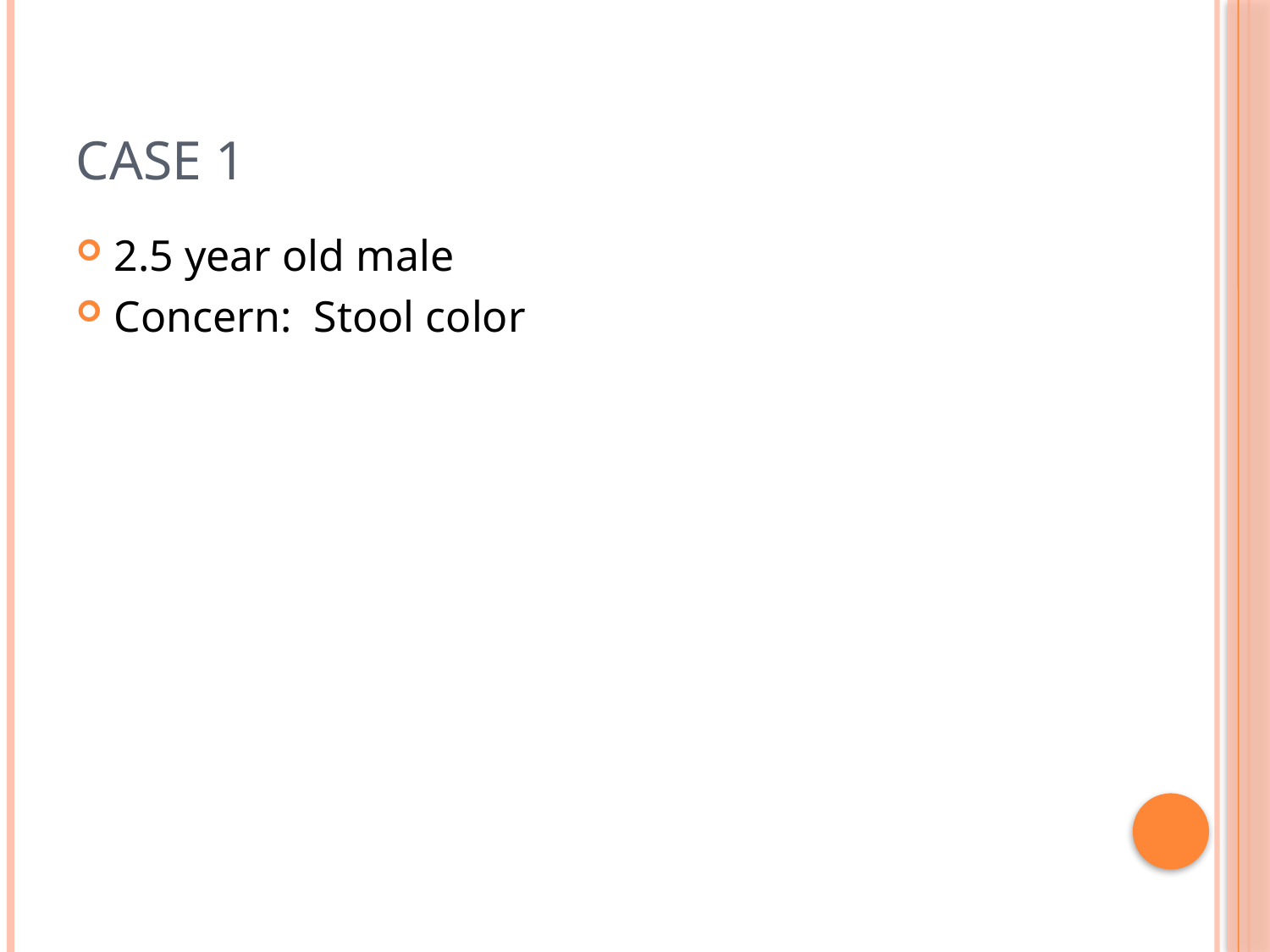

# Case 1
2.5 year old male
Concern: Stool color

## Slide 4
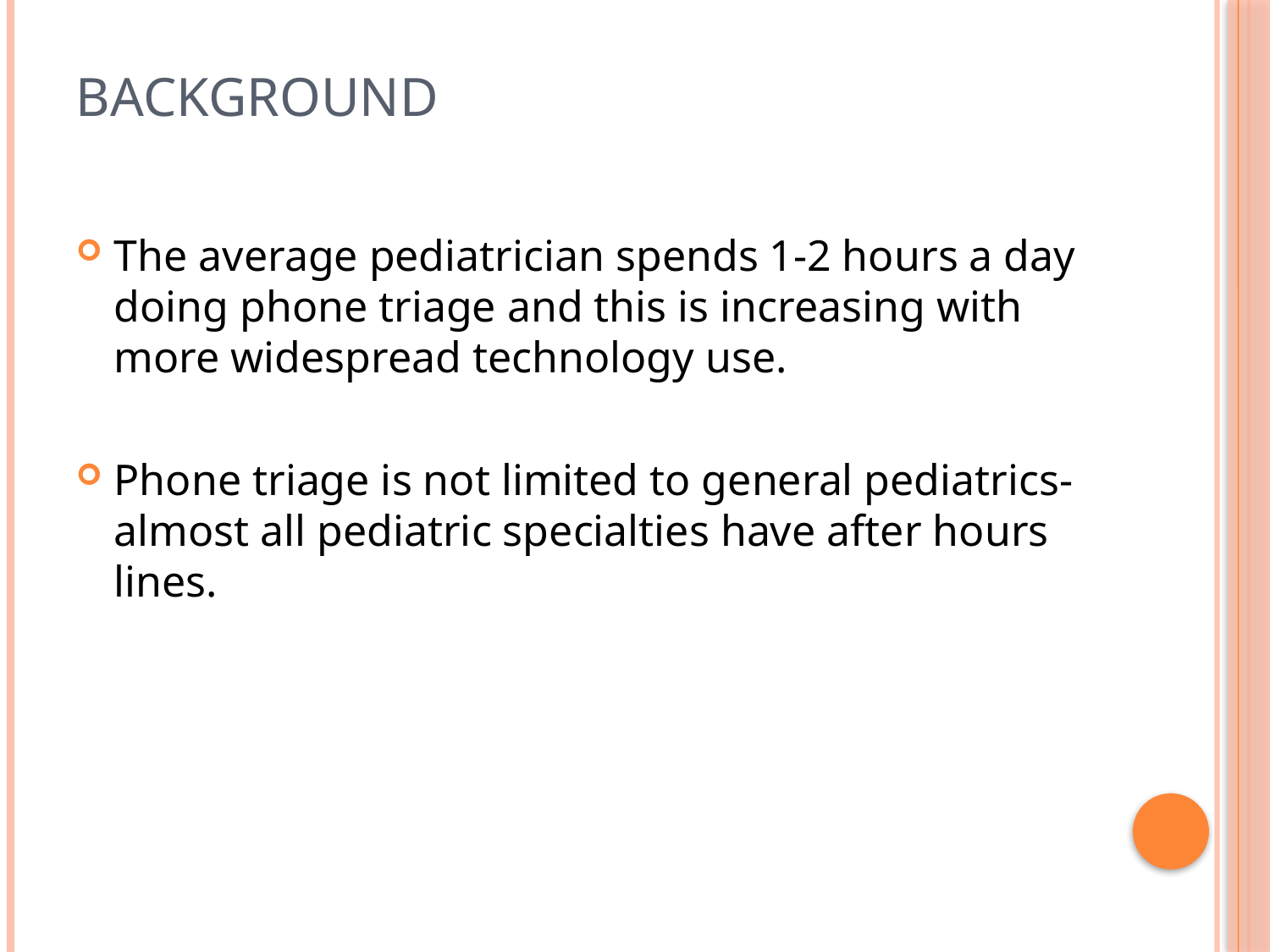

# Background
The average pediatrician spends 1-2 hours a day doing phone triage and this is increasing with more widespread technology use.
Phone triage is not limited to general pediatrics- almost all pediatric specialties have after hours lines.

## Slide 5
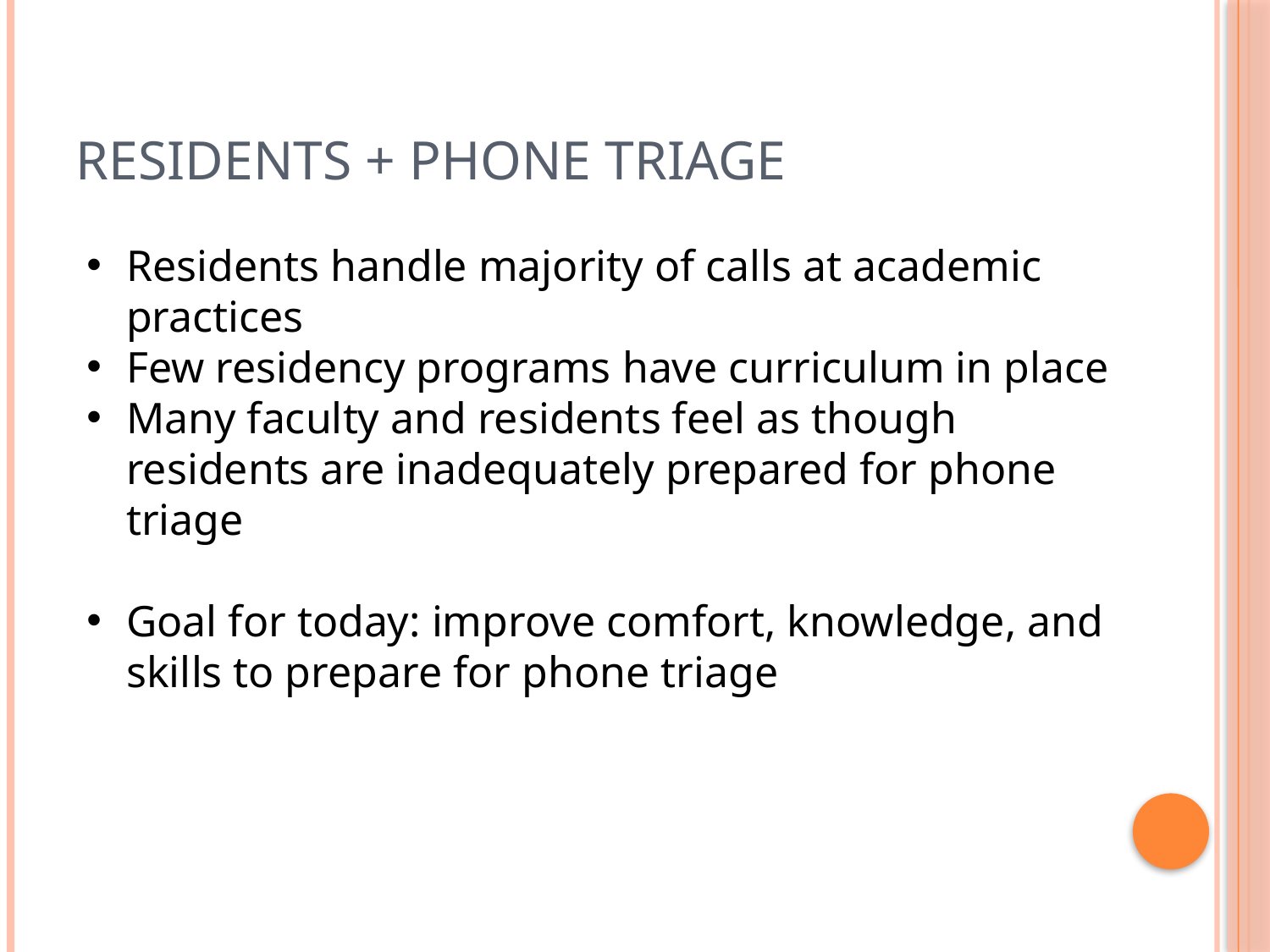

# Residents + phone triage
Residents handle majority of calls at academic practices
Few residency programs have curriculum in place
Many faculty and residents feel as though residents are inadequately prepared for phone triage
Goal for today: improve comfort, knowledge, and skills to prepare for phone triage

## Slide 6
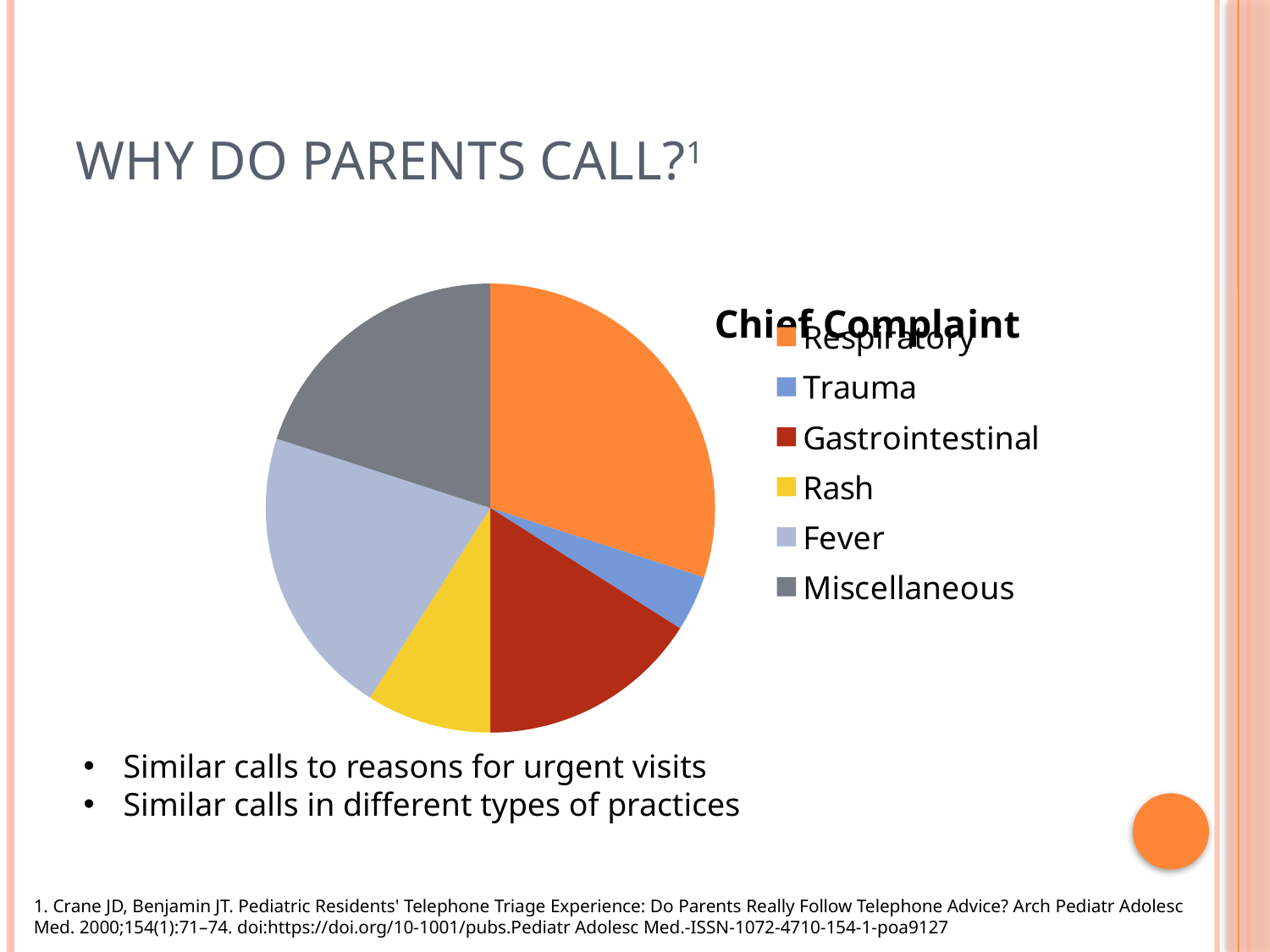

# Why do parents call?1
### Chart:
| Category | Chief Complaint |
|---|---|
| Respiratory | 30.0 |
| Trauma | 4.0 |
| Gastrointestinal | 16.0 |
| Rash | 9.0 |
| Fever | 21.0 |
| Miscellaneous | 20.0 |Similar calls to reasons for urgent visits
Similar calls in different types of practices
1. Crane JD, Benjamin JT. Pediatric Residents' Telephone Triage Experience: Do Parents Really Follow Telephone Advice? Arch Pediatr Adolesc Med. 2000;154(1):71–74. doi:https://doi.org/10-1001/pubs.Pediatr Adolesc Med.-ISSN-1072-4710-154-1-poa9127

## Slide 7
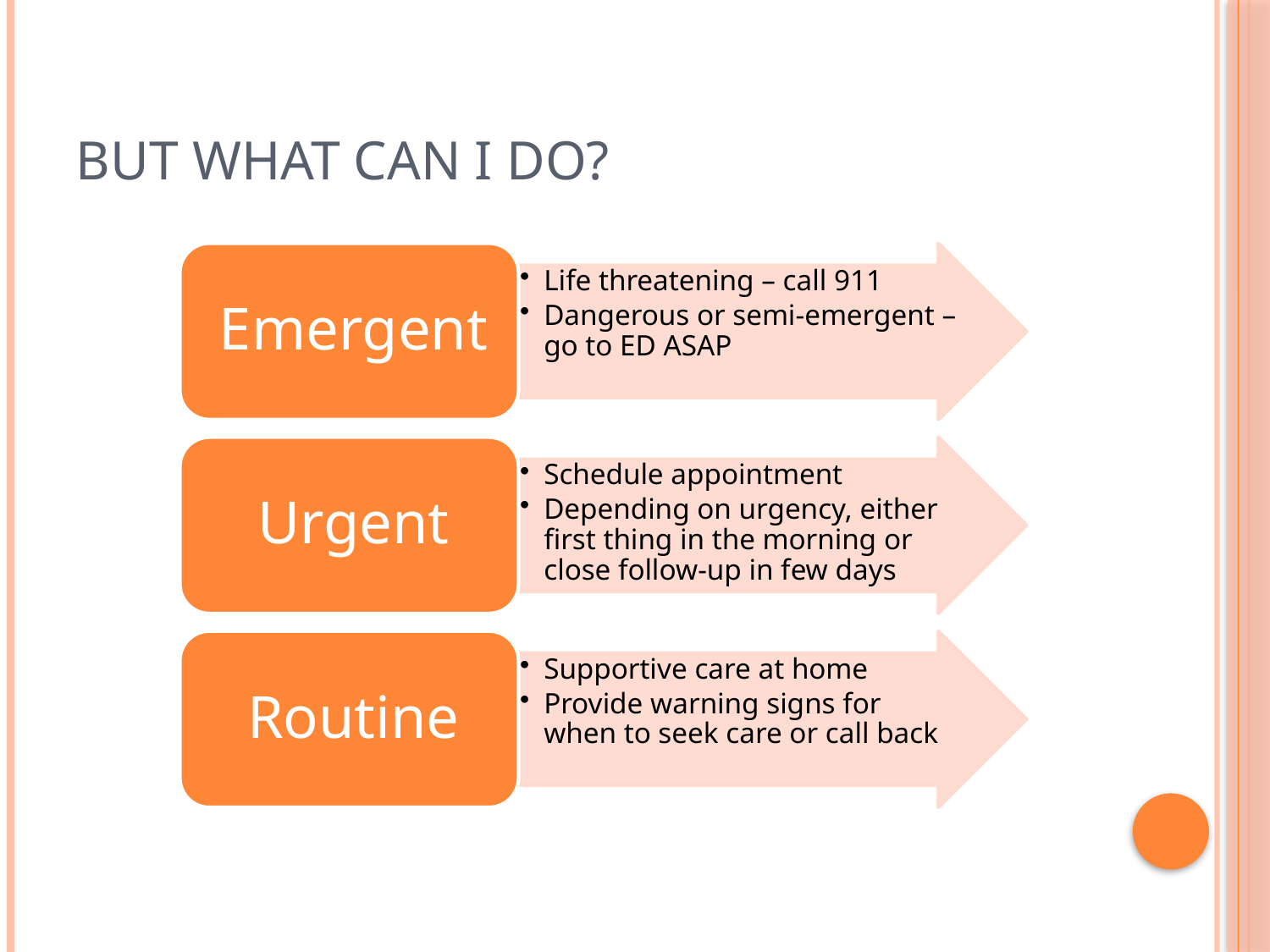

# But what can I do?

## Slide 8
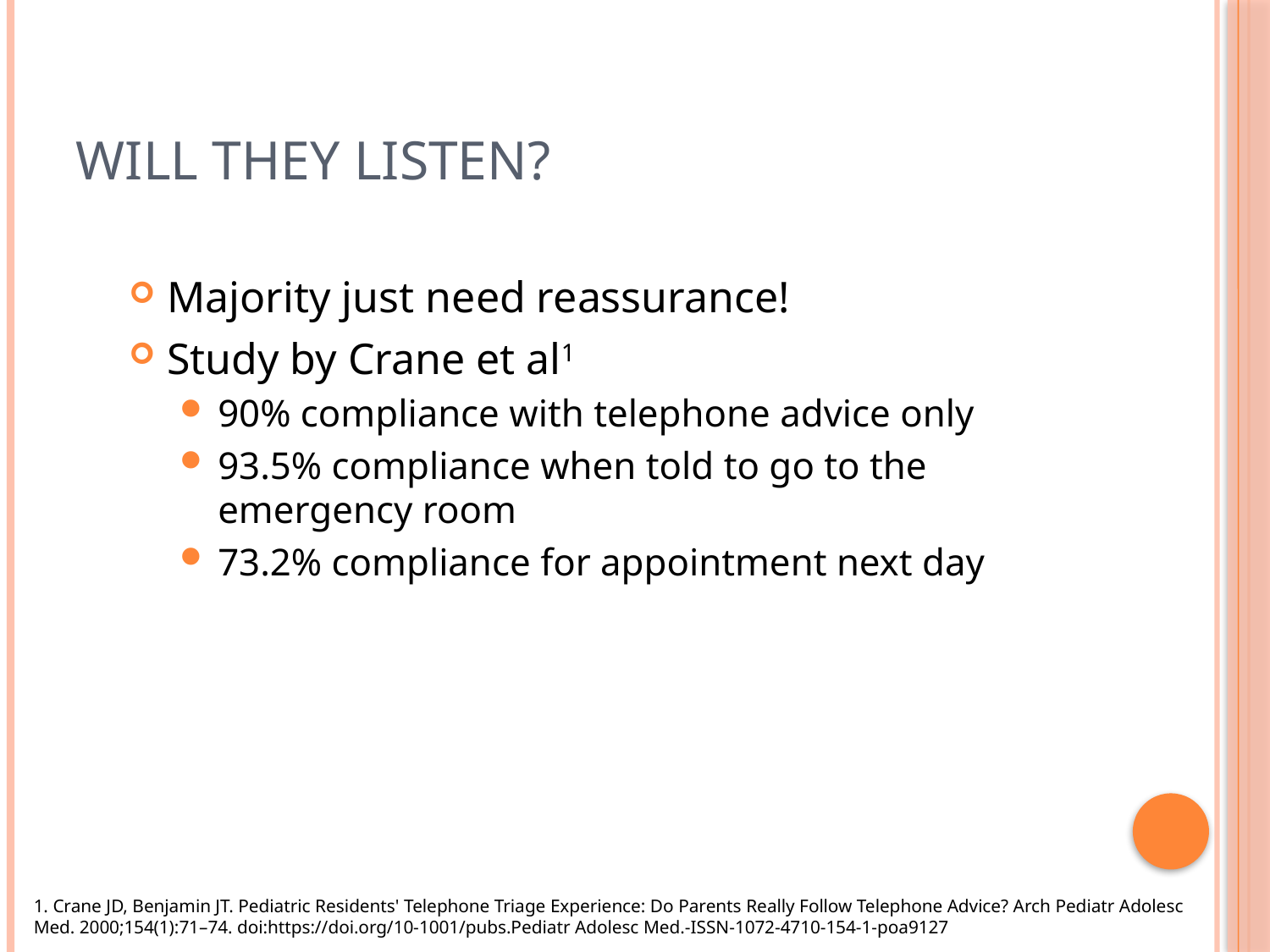

# Will they listen?
Majority just need reassurance!
Study by Crane et al1
90% compliance with telephone advice only
93.5% compliance when told to go to the emergency room
73.2% compliance for appointment next day
1. Crane JD, Benjamin JT. Pediatric Residents' Telephone Triage Experience: Do Parents Really Follow Telephone Advice? Arch Pediatr Adolesc Med. 2000;154(1):71–74. doi:https://doi.org/10-1001/pubs.Pediatr Adolesc Med.-ISSN-1072-4710-154-1-poa9127

## Slide 9
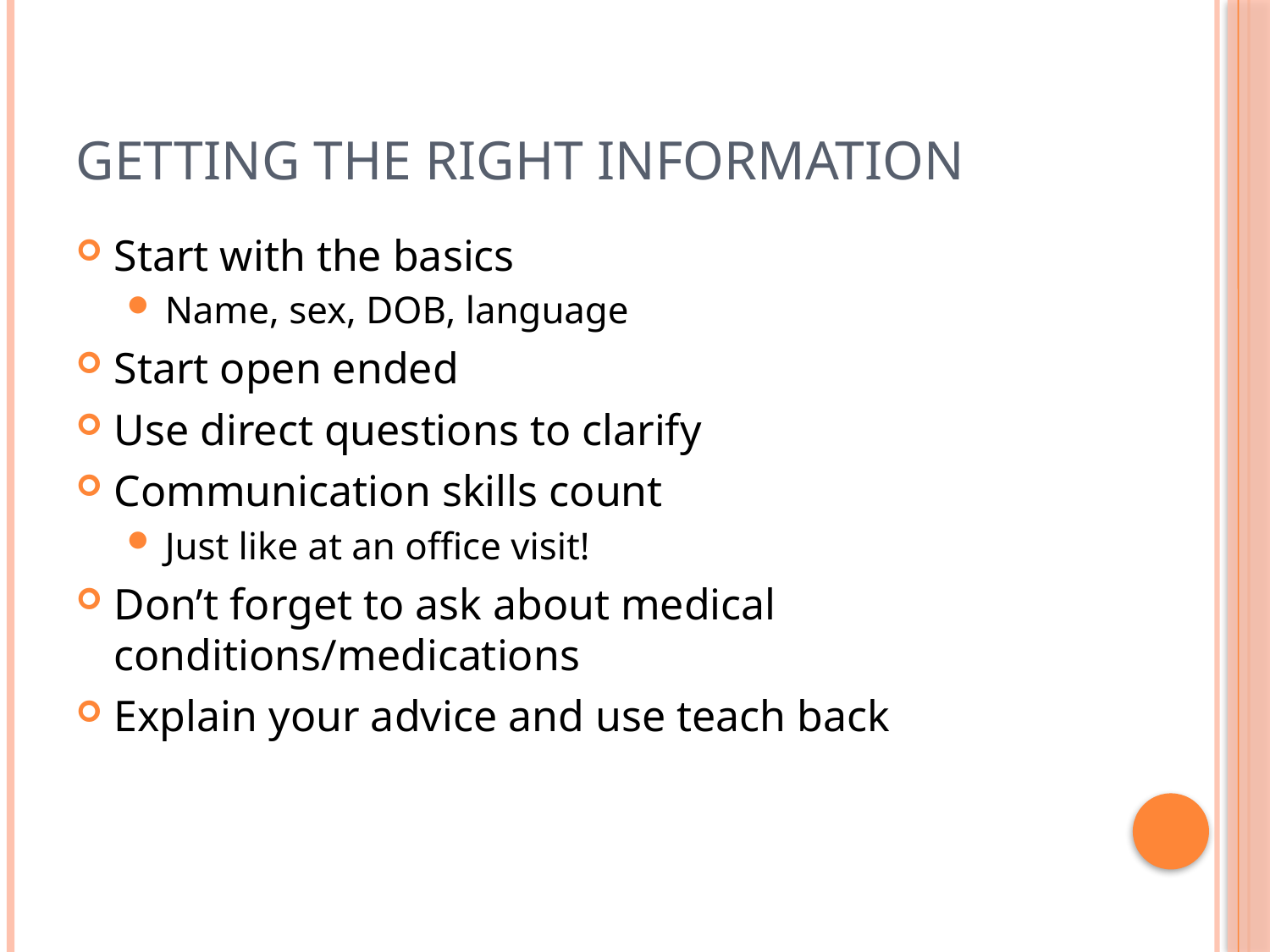

# Getting the right information
Start with the basics
Name, sex, DOB, language
Start open ended
Use direct questions to clarify
Communication skills count
Just like at an office visit!
Don’t forget to ask about medical conditions/medications
Explain your advice and use teach back

## Slide 10
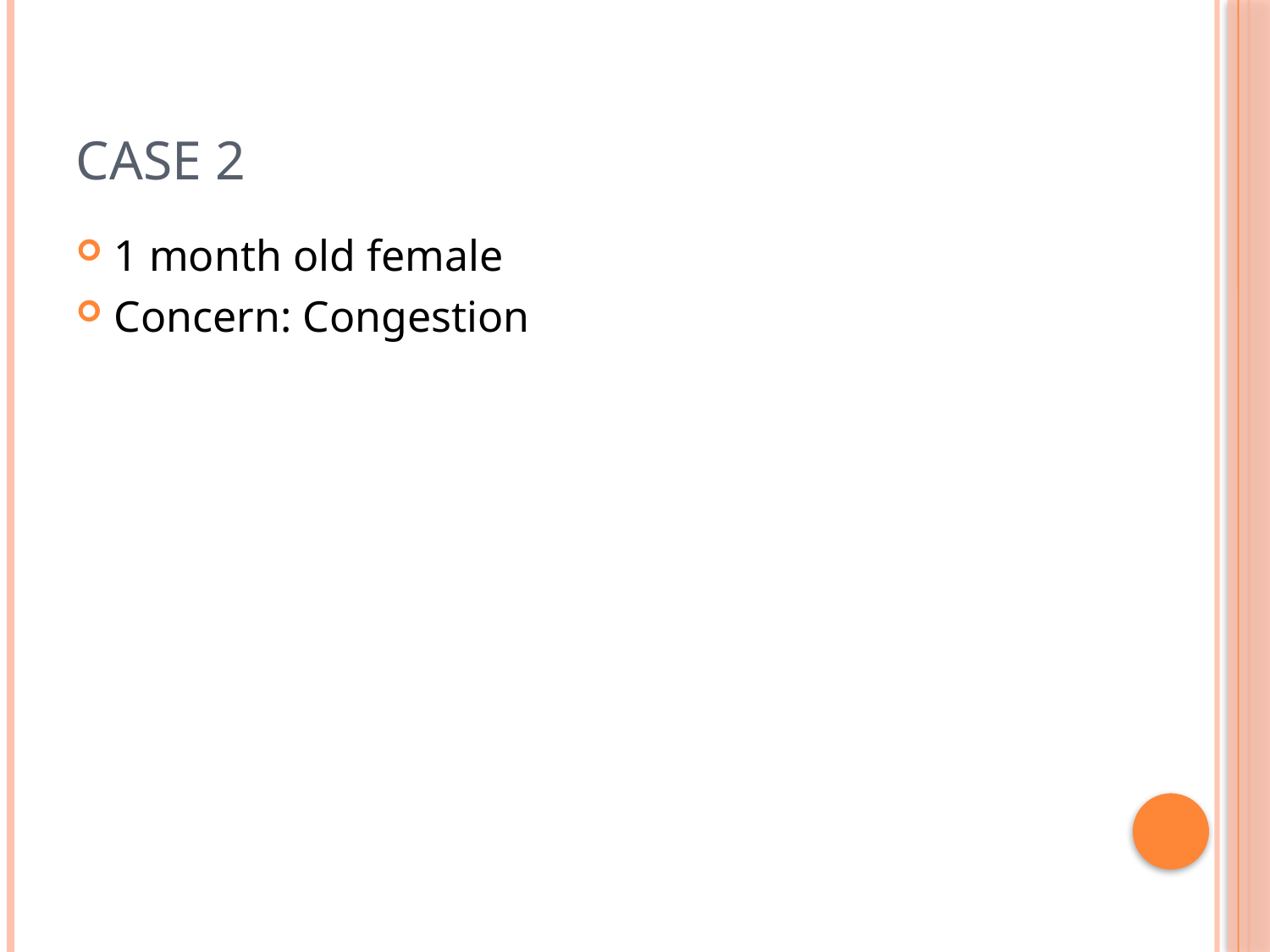

# Case 2
1 month old female
Concern: Congestion

## Slide 11
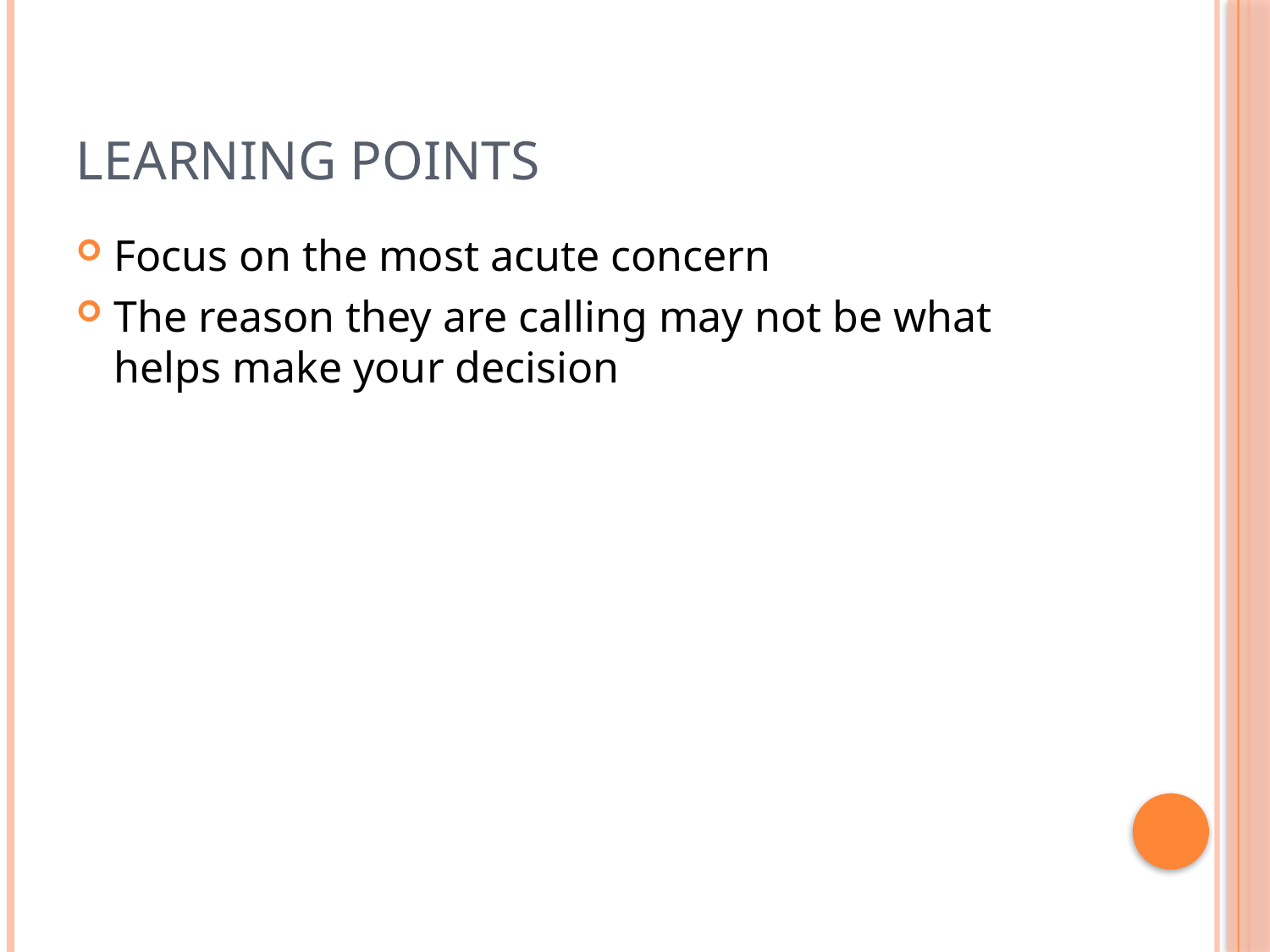

# Learning points
Focus on the most acute concern
The reason they are calling may not be what helps make your decision

## Slide 12
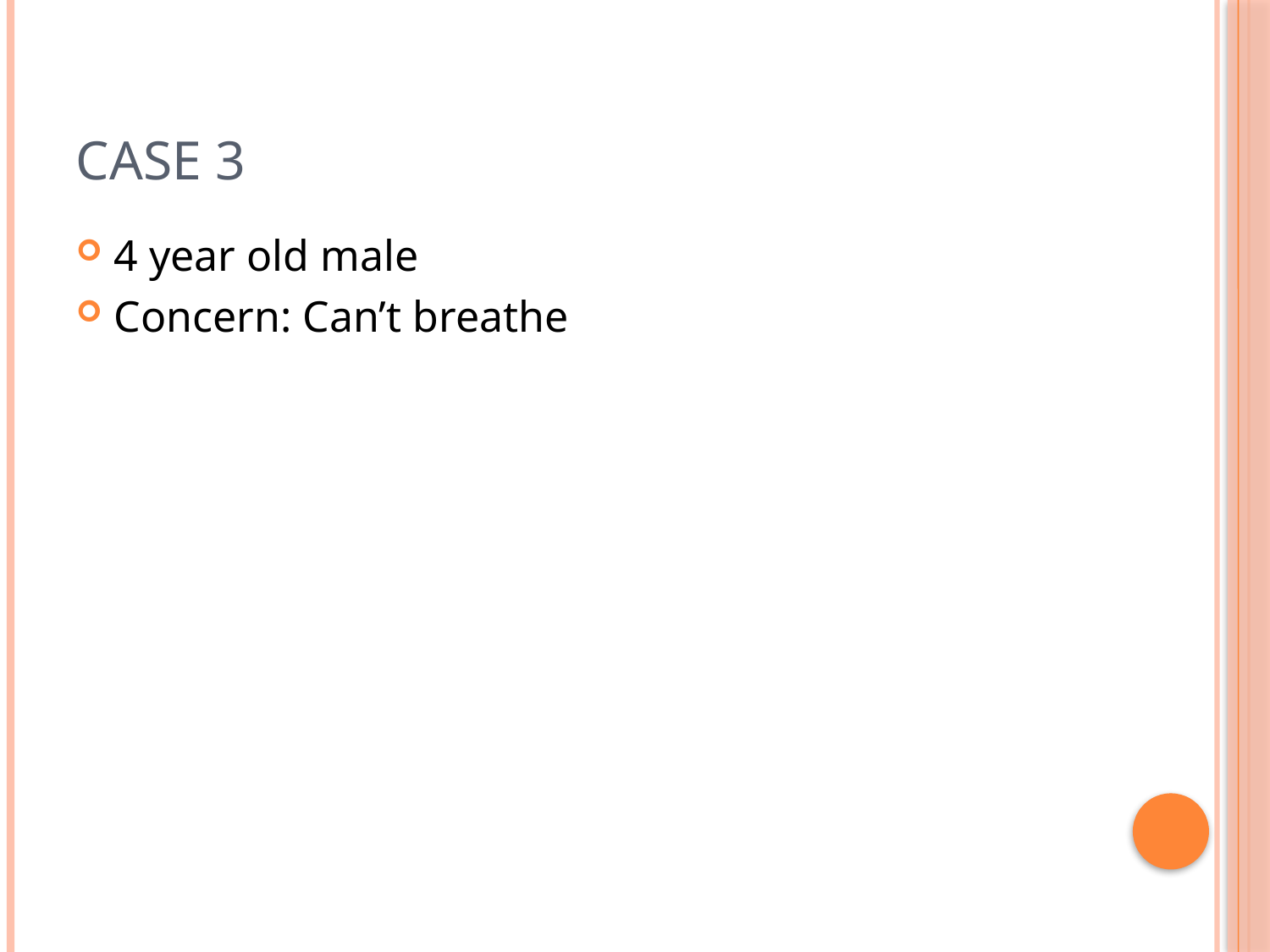

# Case 3
4 year old male
Concern: Can’t breathe

## Slide 13
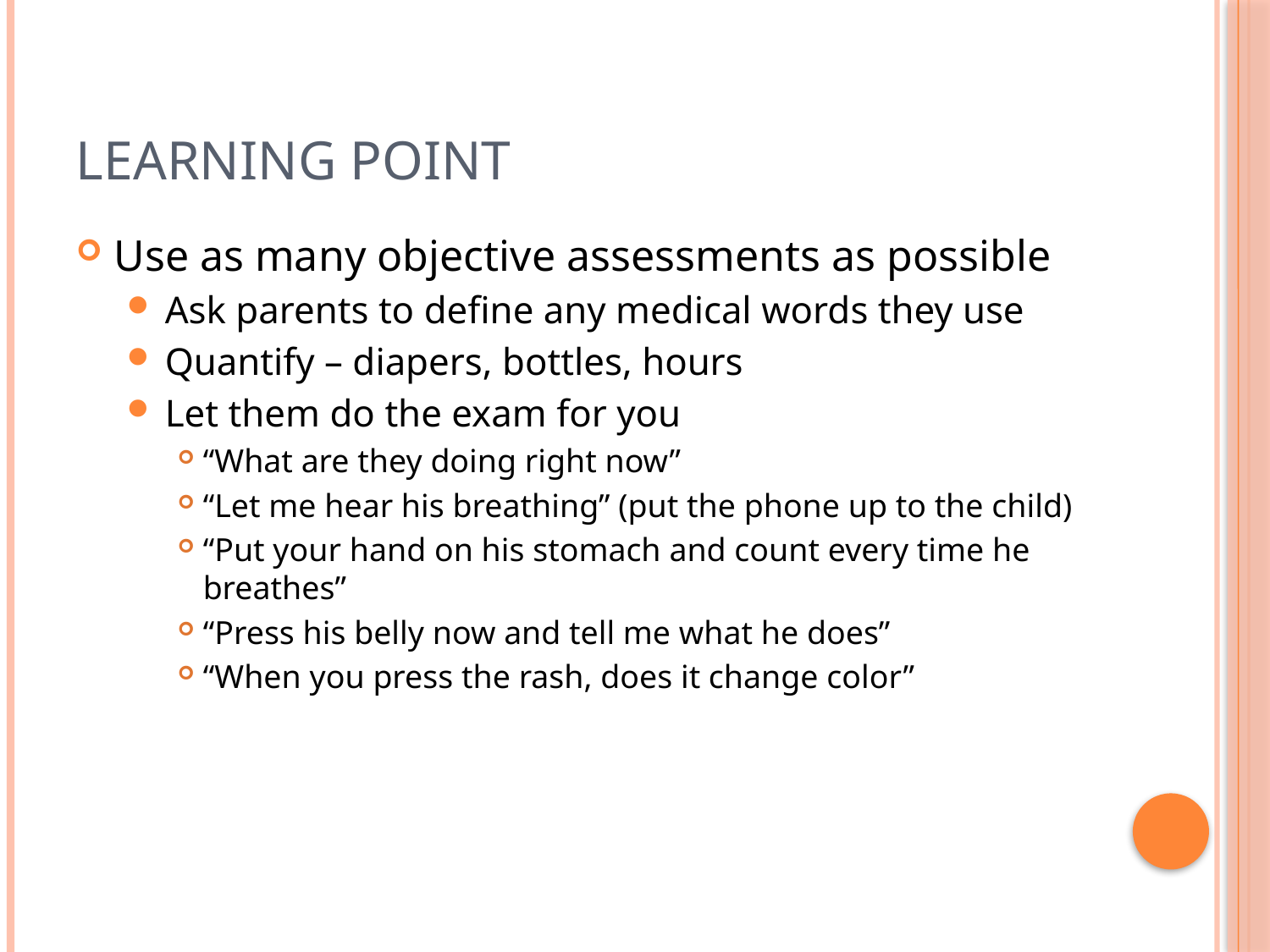

# Learning Point
Use as many objective assessments as possible
Ask parents to define any medical words they use
Quantify – diapers, bottles, hours
Let them do the exam for you
“What are they doing right now”
“Let me hear his breathing” (put the phone up to the child)
“Put your hand on his stomach and count every time he breathes”
“Press his belly now and tell me what he does”
“When you press the rash, does it change color”

## Slide 14
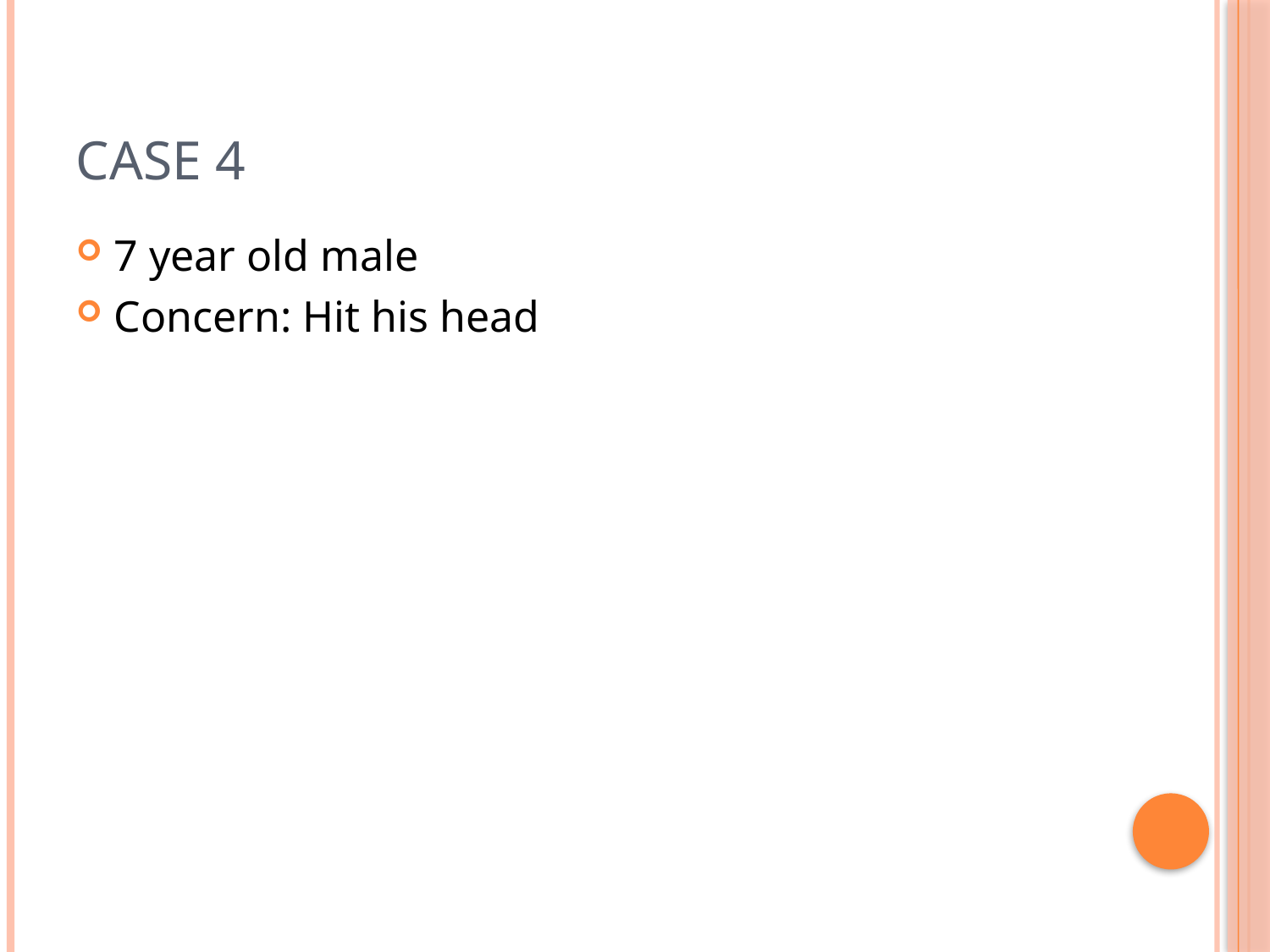

# Case 4
7 year old male
Concern: Hit his head

## Slide 15
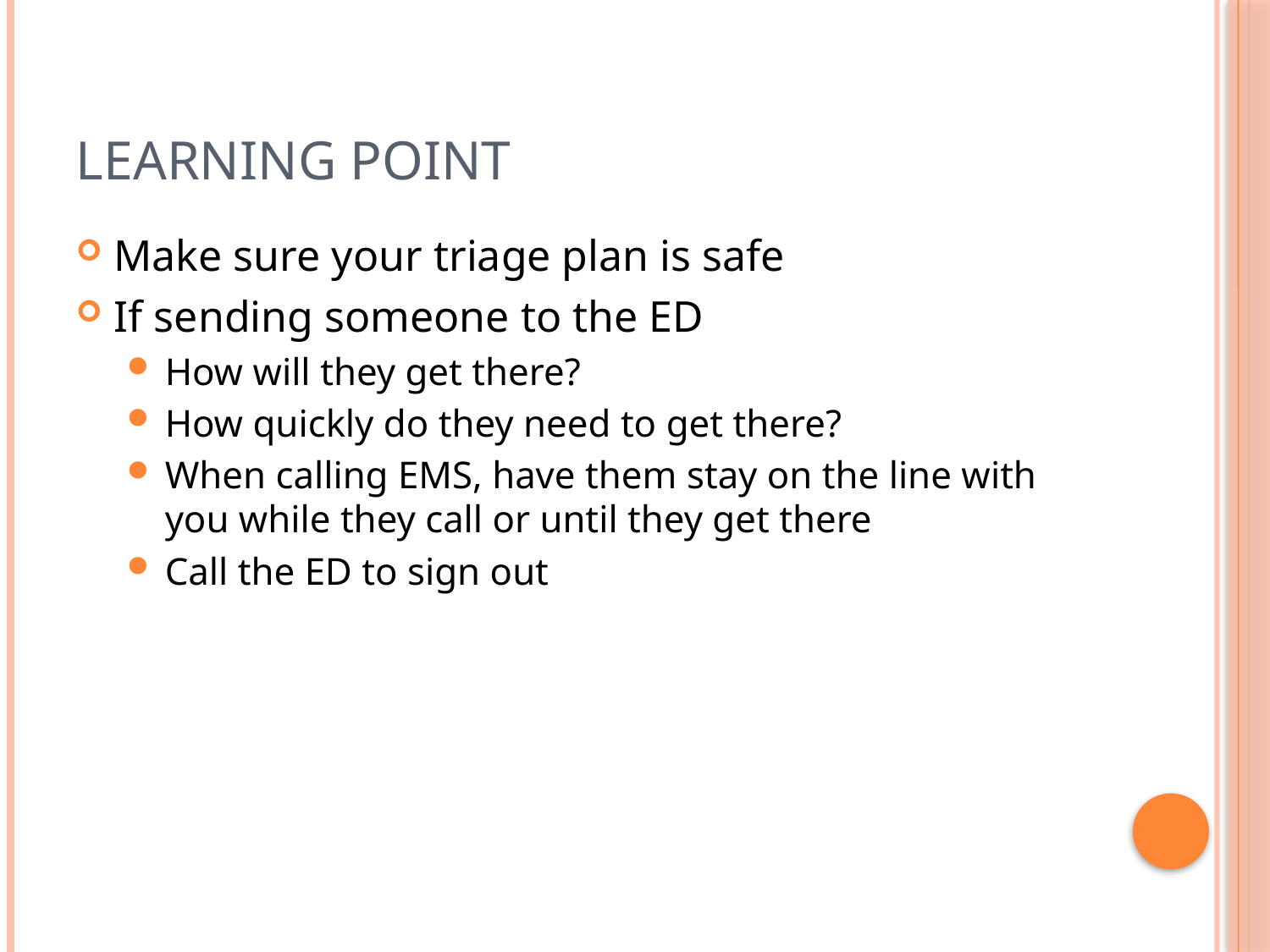

# Learning point
Make sure your triage plan is safe
If sending someone to the ED
How will they get there?
How quickly do they need to get there?
When calling EMS, have them stay on the line with you while they call or until they get there
Call the ED to sign out

## Slide 16
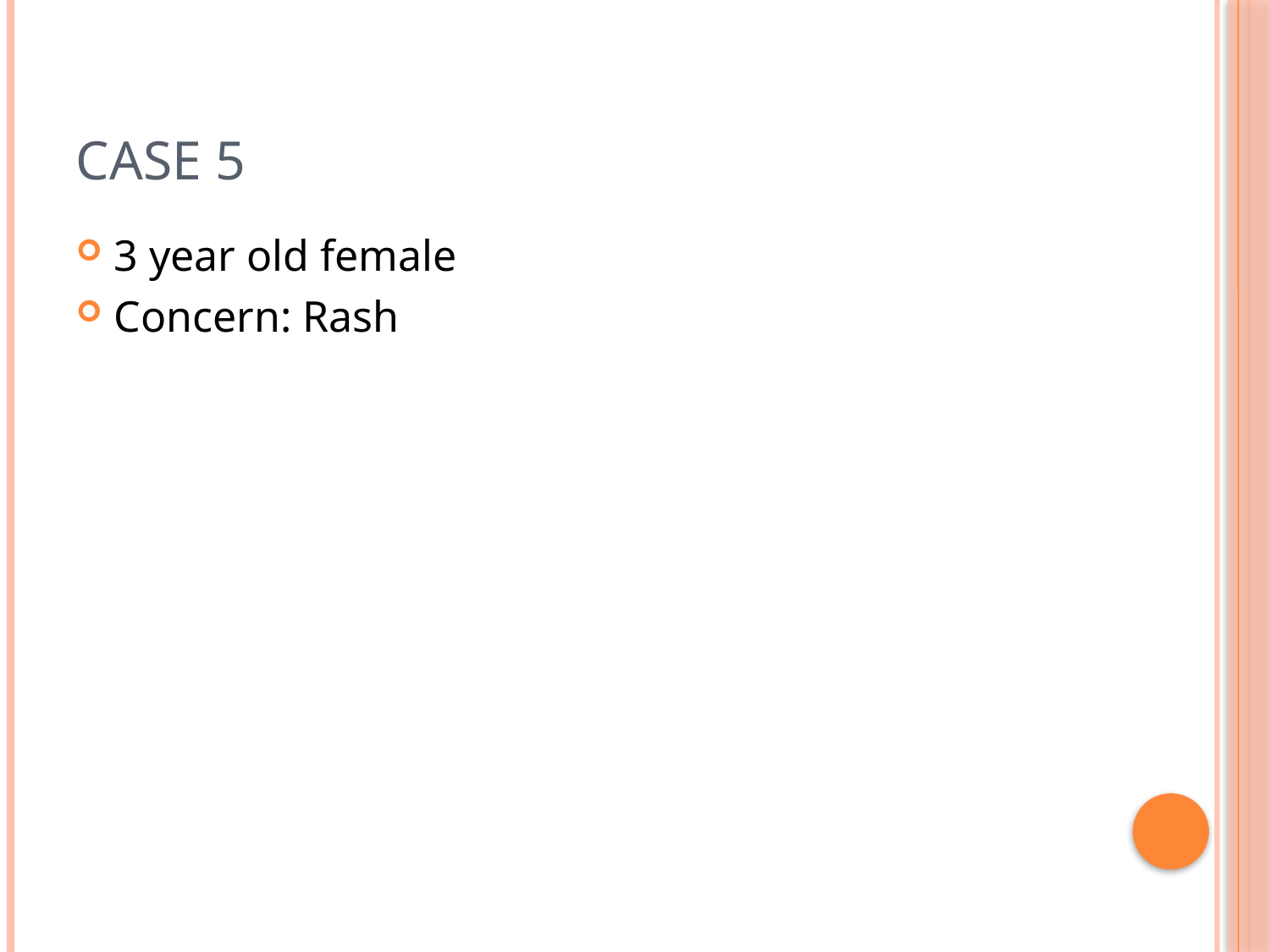

# Case 5
3 year old female
Concern: Rash

## Slide 17
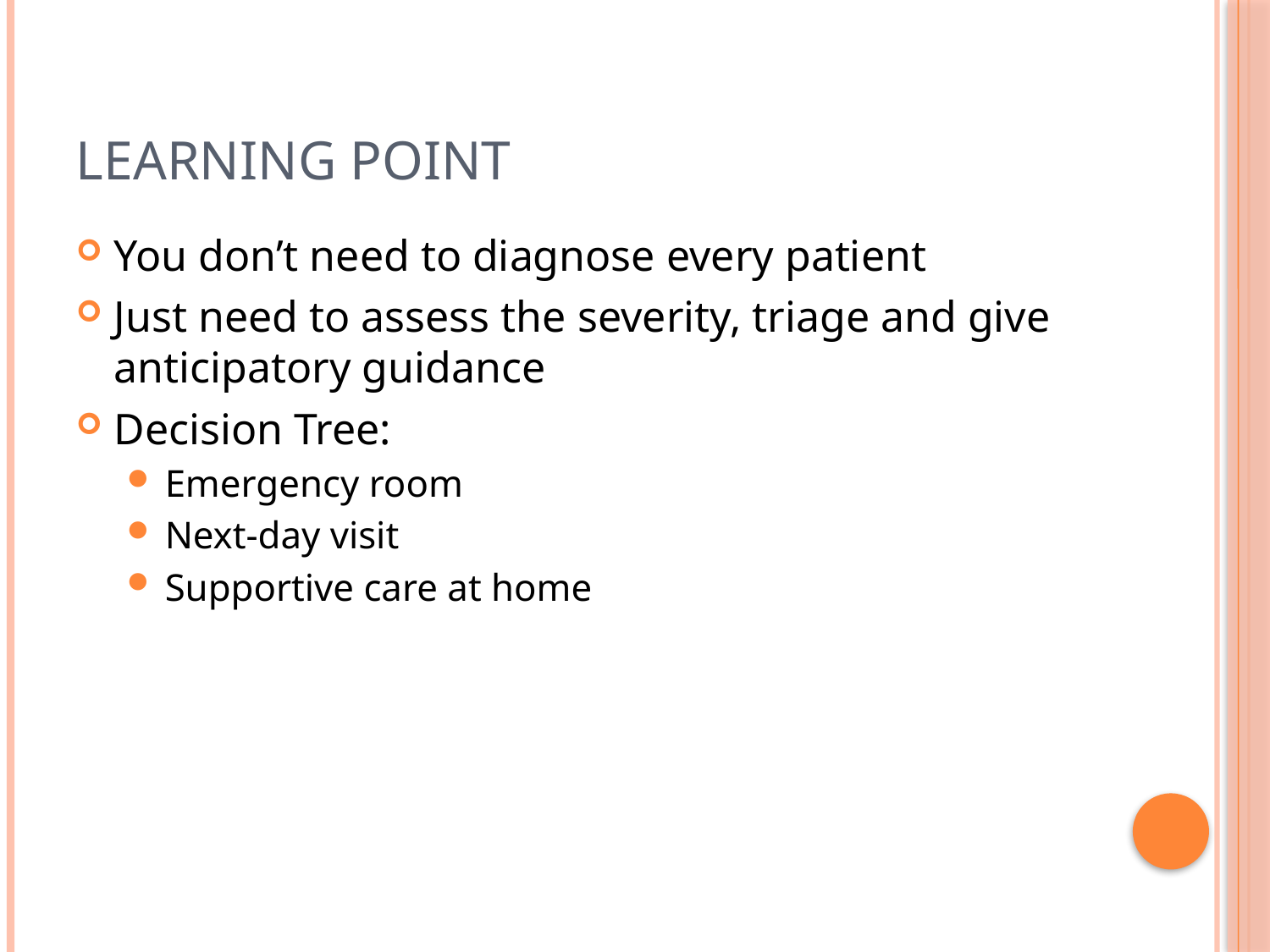

# Learning Point
You don’t need to diagnose every patient
Just need to assess the severity, triage and give anticipatory guidance
Decision Tree:
Emergency room
Next-day visit
Supportive care at home

## Slide 18
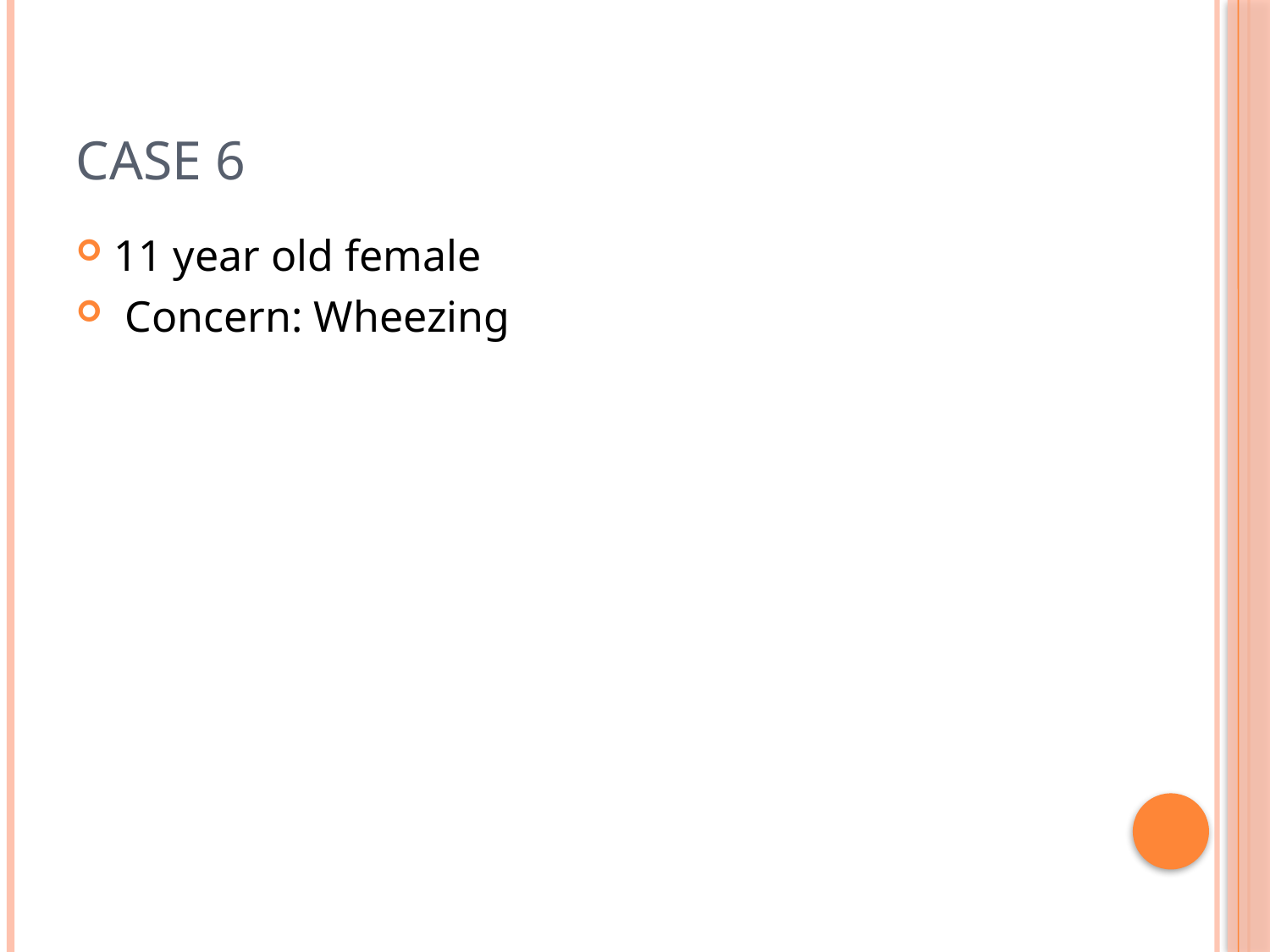

# Case 6
11 year old female
 Concern: Wheezing

## Slide 19
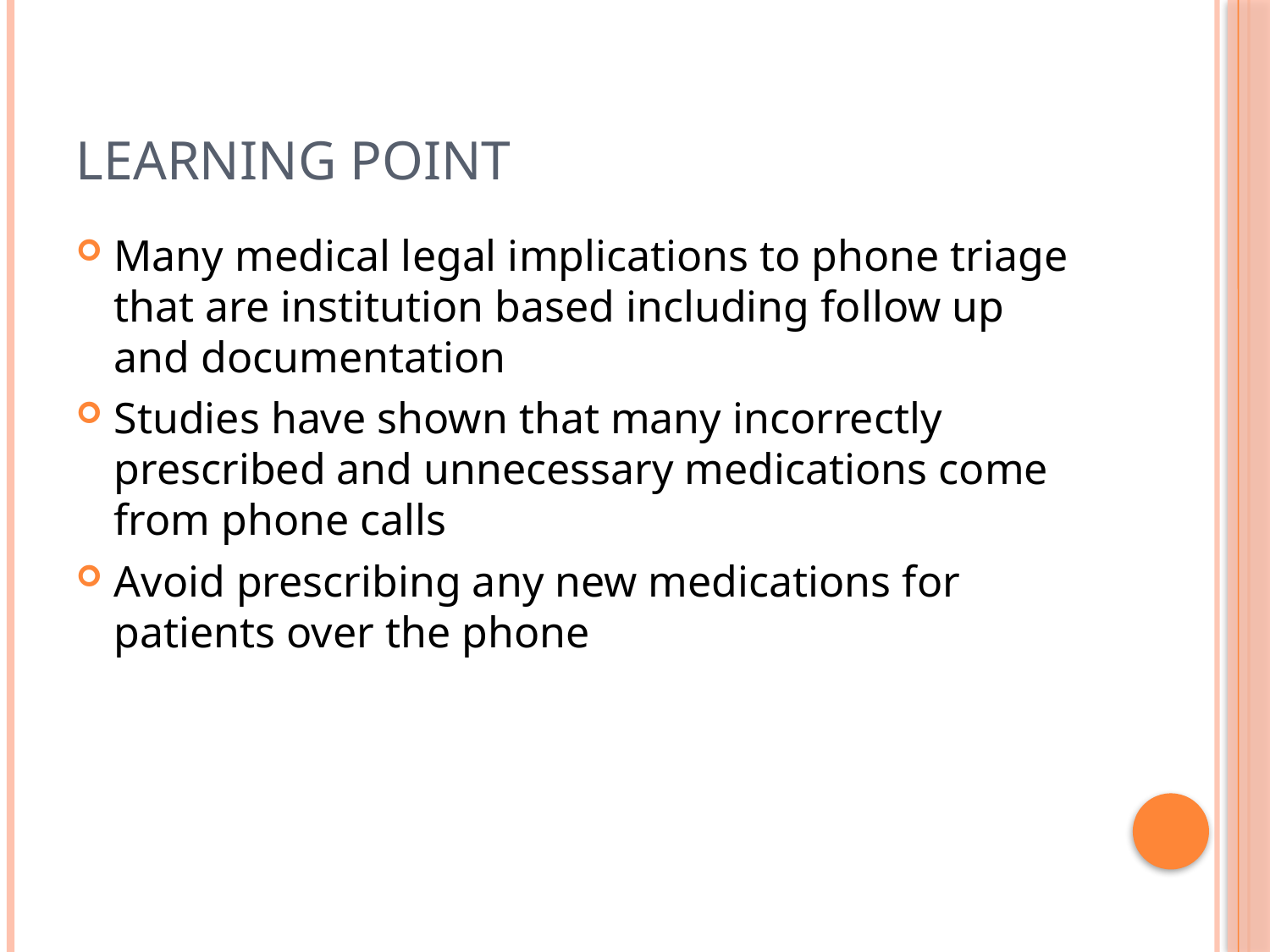

# Learning Point
Many medical legal implications to phone triage that are institution based including follow up and documentation
Studies have shown that many incorrectly prescribed and unnecessary medications come from phone calls
Avoid prescribing any new medications for patients over the phone

## Slide 20
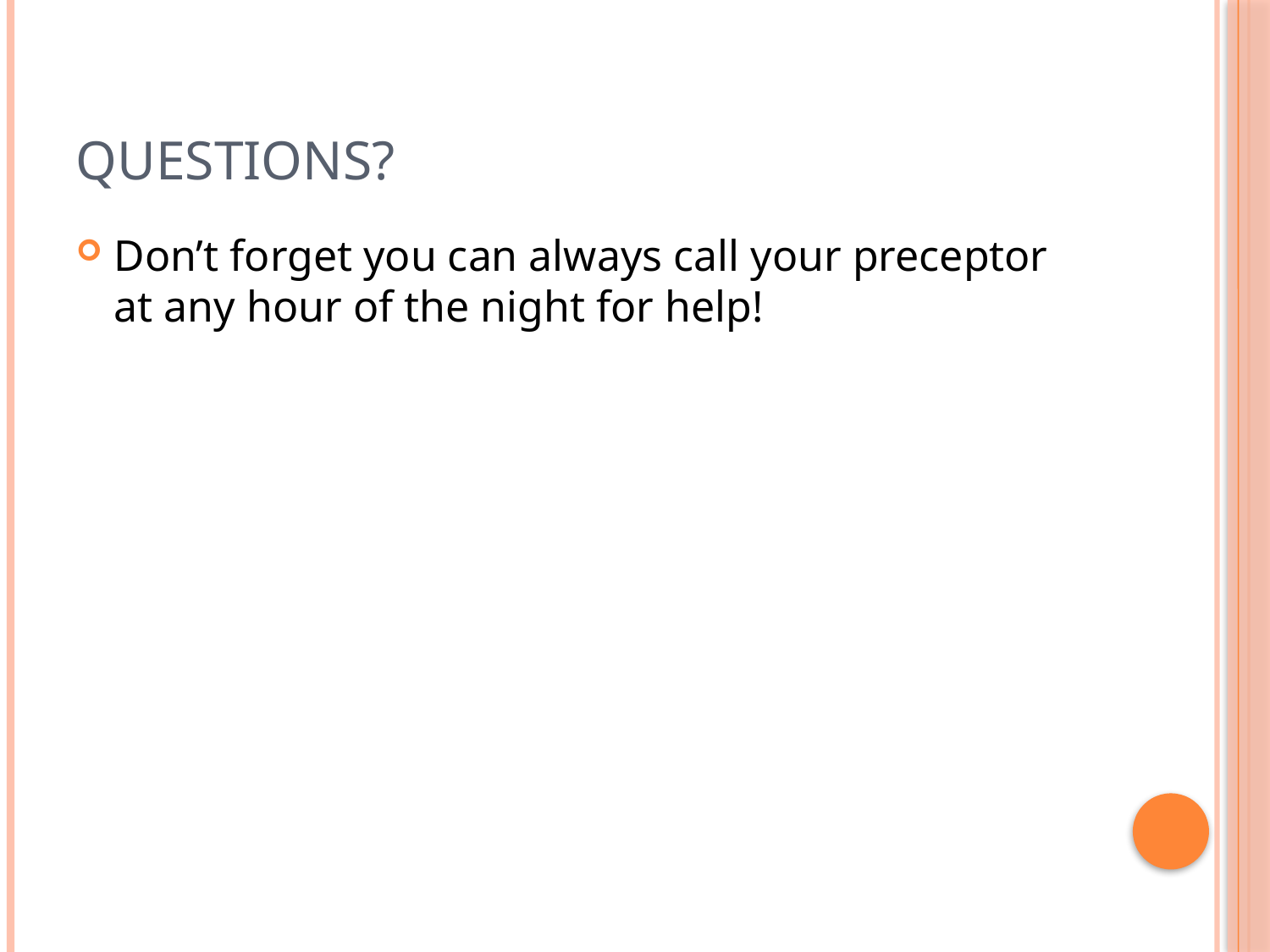

# Questions?
Don’t forget you can always call your preceptor at any hour of the night for help!
