# Supplementary material for: A Curriculum to Improve Pediatric Residents' Telephone Triage Skills
Source: MedEdPORTAL. 2020 Oct 22;16:10993. doi: 10.15766/mep_2374-8265.10993 (PMC7586755; doi:10.15766/mep_2374-8265.10993)
Supplement: Supplementary file 1 — Pediatric Phone Triage Conference Presentation.pptxFaculty Guide - Pediatric Phone Triage Conference.docxJust-in-Time Training.docxResident Cheat Sheet.docxPre- and Postexperience Self-Assessment.docxConvenience Sample Preassessment.docx [file mep_2374-8265.10993-s001.zip › B. Faculty Guide - Pediatric Phone Triage Conference.docx]

**Appendix B: Presentation Faculty Guide**

*This guide follows slide by slide with the presentation for telephone triage. This provides a script that can be used by the facilitator or can just be used as a guide. Case information is given for each of the cases discussed and the facilitator can assign a resident to role play the parent using this information, or do so themselves. It would be optimal to have different residents play the role of doctor for each case to allow for maximal exposure.*

Slide 1: Today we will be discussing telephone triage.

Slide 2: The objectives of today’s talk are to develop the necessary skills to approach telephone triage, discuss techniques that can be used for assessing patients over the phone and practice decision making in common clinical situations that arise.

Slide 3: We will start with a case. One resident can role play the doctor getting this call.

*Provide information for case as it is asked for by the ‘on call- Dr’*

Case 1: The mother of a 2.5-year-old boy calls because she is concerned that his stools have changed color. She has noticed that for the past day his stools have been blue and green in color. This has never happened before. He looks well according to her. He has no abdominal pain, no fevers, he has not traveled. There is no blood in the stool and no vomiting. He got a present of play dough this week and mom is not sure if he has been eating it.

Case debriefing- This case is meant to allow you to practice asking questions over the phone, when you can’t see or examine the patient. Some complaints may be concerning at first but can easily be reasoned through or vice versa. We will discuss some information about telephone triage in general and your role as residents in triage and then continue with more practice cases

Slide 4: Most pediatricians spend a large portion of their day doing phone triage with families and as technology is becoming more widespread, this is expected to increase as well. While some people think of phone triage as a general outpatient pediatrics skill, the majority of pediatric specialties have after-hours lines that are staffed by physicians, fellows or nurses. Regardless of what sub-specialty you ultimately choose, there is a good chance you will need to develop telephone triage skills

Slide 5: At most academic practices, residents are the ones handling the majority of after-hours calls. Currently less than 50% of residency programs have curricula for teaching this skill and most faculty think the residents aren’t prepared, and the residents agree. We hope that by the end of this conference, you will feel more prepared in this skill.

Slide 6: In general, parents call for the same reasons they come in to the office for urgent visits and this is true across many different type of practice sites. The most common calls are for respiratory complaints, fevers and GI issues.

Slide 7: What is different in telephone triage is that you don’t need to diagnose the patient over the phone. The first step is deciding if the patient is experiencing an emergency or not- and if so, how soon they need to be seen. If they are not experiencing an emergency, do they need to be seen by a physician at all. If so, how soon. And then in about 50% of cases, an appointment in unnecessary and home treatment advice and reassurance are all the parent needs. The goal is not a diagnosis, it is appropriate triage to get a diagnosis if needed.

Slide 8: You may wonder if the patients will listen to your advice when they call. Studies show that the majority of parents do listen to the advice of the doctor – especially when it is for telephone advice only.

Slide 9: In order to effectively triage a patient, you need to be able to get the right information from them. To start with you need the basics including their name, age and language preference. Just like in office visits your communication skills are key. Start with open ended questions to allow them to explain their reason for calling and use direct questions for clarifications and to get more details. Since you may not have their medical information in front of you, make sure to ask about medical conditions and medications. At the end of the call, allow time for questions, explain your advice and use teach back to make sure directions are clear

Slide 10: Lets practice with another case

*Provide information for case as it is asked for by the ‘on call- Dr’*

Case 2: The mother of a 1 month old calls with the chief complaint of congestion. She is very concerned that the baby is congested and can’t breathe. She has tried suctioning but it isn’t helping and she thinks the baby is breathing faster than they should and even pauses for a few seconds at a time when she breathes. She is coughing and not eating as well as usual. She felt warm and mom took her temperature and it was 100.8F. She is not vomiting or having diarrhea and has no sick contacts. When you ask mom to count her breathing she is breathing at 30 and not using any intercostal muscles or retracting.

*If the resident gets all of the information they should triage them as an emergency and send them to the ED for a rule out sepsis workup for fever in a neonate. If the resident does not get all of the information since the patient’s complaint was congestion, they may reassure them about normal infant congestion and periodic breathing. In this case, give them the full history prior to moving on to the learning points of the case.*

Slide 11: This call could have been very different based on the chief complaint of the parent. It is important to ask about acute concerns related to the complaint because the reason the parent is calling may not be what makes you decide if they are sick or not.

Slide 12: *Provide information for case as it is asked for by the ‘on call- Dr’*

Case 3: The mother of a 4-year-old boy calls with the complaint that he can’t breathe. The mom reports that he has been sick with a cold all week and has been getting worse. She is very worried and wants to take him the ED but was calling you first to let you know. He has a fever to 101.2, has been coughing and congested. He doesn’t have any past medical history and the only medication she gave him was ibuprofen for the fever. He isn’t eating well but his urine output has been normal. He is currently sitting and watching TV. He is comfortable but holding his mouth open to breathe. She counts his breathing rate at 20 and he is not using any intercostal muscles or retracting.

*If the resident gets all of the information they should provide supportive care at home (with the option of an office visit the following day) with explanation of the reasons to call back, bring the child in or take him to the ED. If they do not get to the ‘objective’ findings, they may recommend sending the patient to the ED because of the parent’s report that the patient can’t breathe. In this case, give them the full history prior to moving on to the learning points of the case.*

Slide 13: When you aren’t able to see a patient, it can often be hard to assess how sick they are. When taking a history over the phone, it is important to use as many objective assessments as possible to replace the fact that you aren’t with the patient. If the parent uses any medical terminology (for example: my daughter is wheezing), ask them to explain what they mean by that as opposed to taking it at face value. Ask them to quantify what they tell you (for example- 8 diapers today as opposed to urinating normally). When you can, let the parent do the exam for you. This can be as simple as asking what the patient looks like and what they are doing right now to having the parent perform exam maneuvers such as counting their breathing rate, touching their stomach or putting them up to the phone so you can hear them.

Slide 14: *Provide information for case as it is asked for by the ‘on call- Dr’*

Case 4: The mom of a 7-year-old boy calls because he was playing with his brother and he jumped from the table to the couch and hit his head on the corner of the couch and fell to the floor. He cried immediately and didn’t lose consciousness. Since then he has vomited twice. She wants to know if you think he has a concussion because he is acting sleepy and confused and not really making sense. When she touches his head he says it hurts and he has a big red bruise on the side of his head but no bleeding. She tried putting ice on the bump and now just wants to put him to bed.

*If the resident gets all of the information they should recommend this patient go to the emergency room for evaluation given his vomiting and altered mental status after a head injury. If they do not get all of the information, they may offer supportive care. In this case, give them the full history prior to moving on to the learning points of the case.*

Slide 15: In this case, although the mother thought the patient could go to sleep to help his injury, you likely recognized that he had some concerning signs of a head injury based on the PECARN guidelines (Kupperman et al) and recommended he go to the emergency room. In these cases, you also need to think about if your triage plan is safe. If you are sending someone to the ED, how do you want them to get there? Is this someone who needs to get there immediately, in which case you may want to call EMS or can the parent take them? Is the parent reliable and invested enough in the plan to get them to the ED? Are you concerned that if the parent takes them they may decompensate on the way? Once you have triaged a patient to an emergency, you will need to go the step further and decide how quickly and what is the best way for them to get emergency services. In addition, depending on your hospital set up, you may want to call the ED to alert them that the patient is on the way. *You can give the residents the specifics for your local practices.*

Slide 16: *Provide information for case as it is asked for by the ‘on call- Dr’*

Case 5: The father of a 3-year-old girl calls concerned about a rash she has had for the past day. He doesn’t think she has eaten anything new or used any new products. She hasn’t been playing outside at all and no one else in the family has it. She isn’t particularly bothered by it and says it’s not hurting or itching her. She has some congestion but no cough, shortness of breath or wheezing. She has no fevers. He describes her as looking very comfortable but with a red bumpy rash.

*If the resident gets all of the information, they should recommend supportive care or a visit within the next few days for an exam. If they do not get all of the information, give them the full history prior to moving on to the learning points of the case.*

Slide 17: The differential on what may be the cause of this patient’s rash can be quite broad. However, unlike at a visit where you may want to determine the exact etiology, as much as possible, over the phone the important factor is the severity of illness and if and when the patient needs to be seen. Going back to this image we reviewed at the beginning, the first step is deciding if this is an emergency or not. In this case, you would want to ask questions to discern that such as if the patient is having a rash consistent with an anaphylactic reaction and needs emergency care. If not, providing advice, anticipatory guidance and the option of an appointment is sufficient.

Slide 18: *Provide information for case as it is asked for by the ‘on call- Dr’*

Case 6: The home nurse of an 11-year-old F with multiple chronic medical conditions including asthma calls to tell you that she thinks she is wheezing. She has been giving albuterol every 4 hours but has not been responding well to it, though her breathing seems to improve for a short time. She has a fever, a cough, congestion. She has had this before as she typically gets asthma symptoms whenever she gets a cold and she wants you to prescribe steroids so she can avoid going to the doctor. Her breathing is not labored, she is not breathing faster than usual after the albuterol and is not using any accessory muscles. Her nurse is able to take her o2 at home and it is normal.

Slide 19: In this case, the patient’s nurse is calling asking for a medication for what seems like an asthma exacerbation, that she recognizes because of the patient’s history. There are many medical-legal implications to phone triage in general and especially prescribing medications over the phone. Studies have shown that when medications are prescribed over the phone, many incorrect and unnecessary prescriptions result and as such many practitioners do not prescribe any new medications during a phone triage call. *Discuss the rules at your institution including new prescriptions, renewing medications and documentation of visits at this point.*
