# Supplementary material for: A Curriculum to Improve Pediatric Residents' Telephone Triage Skills
Source: MedEdPORTAL. 2020 Oct 22;16:10993. doi: 10.15766/mep_2374-8265.10993 (PMC7586755; doi:10.15766/mep_2374-8265.10993)
Supplement: Supplementary file 1 — Pediatric Phone Triage Conference Presentation.pptxFaculty Guide - Pediatric Phone Triage Conference.docxJust-in-Time Training.docxResident Cheat Sheet.docxPre- and Postexperience Self-Assessment.docxConvenience Sample Preassessment.docx [file mep_2374-8265.10993-s001.zip › C. Just-in-Time Training.docx]

Telephone Triage Curriculum

“Just-In-Time” Training

**Objectives for Telephone Triage Curriculum**

At the completion of this curriculum, learners will be able to:

1. Take a complete and appropriate patient history with all essential clinical information from a family member over the phone
2. Effectively triage patients’ clinical needs based on common presentations and red flags in the history they have taken over the phone
3. Improve confidence in autonomous decision-making skills while performing telephone triage without direct supervision

**Goals for “Just-In-Time” Training**

- Review logistics for the on-call system
- Develop skills to approach telephone triage
- Discuss techniques for assessing patient histories and exams over the phone
- Practice decision-making in common clinical situations

**Logistics**

*This information will be tailored to your practice site. Please complete and review prior to the first call:*

- Call Hours: _____________
- How you will be contacted by the call center: ___________________________________
- How to respond to a page: __________________________________________________
- Documentation: __________________________________________________________
- Methods for scheduling appointments for urgent visits: ___________________________
- Rules for medication refills: _________________________________________________
- *Note: avoid calling the patient directly from your cell phone; use a public phone or block the phone number*

**Phone Triage Techniques**

*These techniques are meant to be reviewed as a question and answer session. Residents should feel comfortable with the answers to each of the following questions*

**I: Do I call my attending after each phone call?**

- It is important to contact the attending you are on call with prior to the start of the call to discuss preferences for calling and frequency (i.e. after each patient vs. summarize in the morning).
- Regardless of this set plan, **if you are ever concerned about a patient or have a question, you can always call and ask your attending!**

**II. What baseline information should I ask every patient?**

- Name, date of birth, primary care physician, and chief complaint
- Patient Language – make sure to ask the call center operator what the parent’s preferred language is; if needed, can use the interpreter system to call the patient

**III: What are some of the most common chief complaints/reasons for calling?**

- Fever
- Anti-pyretic dosing
- Cough/cold symptoms
- Pharmacy with questions about prescription done during the day
- Difficulty breathing

**IV: What components of the HPI can you use to triage a patient? (i.e. determine if they are sick and need escalation of care vs. not sick and can follow-up during routine hours)**

- Most acute symptoms (these may not necessarily be their chief complaint), such as difficulty breathing, lethargy, dehydration, persistent high fevers
- Ask specific questions to help elicit acuity (last time they ate, how many wet diapers, last medication administration)
- Past medical history
- Current medications
- What is the parent most concerned about? What made them call in as opposed to waiting until the morning?

**V: What components of the physical exam can you elicit over the phone to assess the patient’s overall condition without seeing them?**

- *General appearance* – What is the child doing right now? Is the child behaving normally or different from baseline?
- *Respiratory exam* – Can you see his ribs when he breathes? Is he breathing much harder or faster than usual? Parents can put their hands on the child’s abdomen/chest and count breaths per minute. If audible stridor or wheezing can hold the phone up to the child.
- *Abdominal exam* – Is his belly rounder or more full than usual? Does it feel soft or firm? Will he let you touch his belly or does he cry out in pain? Which side hurts the most?
- *Hydration status* – Do his lips look dry? Is he crying with tears?
- *Dermatologic exam* – What part of the body is involved? Does the rash seem raised or flat? What does it feel like? Does the color change when you press down on it?

**VI: What if I am not sure what the diagnosis is?**

- You often CANNOT make a definitive diagnosis over the phone and that is okay! You just need to make enough of an assessment to decide **severity of illness,** which will help guide your management plan.

**VII: What additional resources can I use at home?**

- Lexicomp for medication dosing
- Poison control if concerned about an ingestion
- Up-to-date for quick overview of common complaints
- Any additional evidence-based journals or guidelines

**VIII: What are my options in term of management?**

- Emergent – go to the emergency room now. Based on the severity of illness, family comfort and resources, you will need to help them decide if they can go on their own or should call 911 for EMS
- Urgent – advise them to come in for an urgent appointment the following day with warning signs of when to go to the emergency room if any change before their appointment
- Routine/Supportive Care – provide reassurance with warning signs of when to call back or bring the patient in for an appointment; provide anticipatory guidance for what to expect overnight as well as throughout the illness

**IX: What techniques can you use to make sure the family is on the same page as you?**

- Assess the parents’ understanding of the illness/plan
- Use the teach-back method, particularly regarding warning signs
- Ask directly if they are comfortable and agree with the plan you just made for them
- Make sure they know they can always call back if there is any change or they have any questions/concerns

**Practice Cases**

*The following cases are meant to promote discussion. All cases provide a general framework for some of the most common chief complaints. They should be practiced via simulation/role playing. Each case has a checklist of relevant features that each resident should assess to complete each case.*

1. **A parent calls saying her 6 month old feels warm and has been sleeping a lot today. She wants to know if she should take her to the emergency room.**

- Is the child febrile? How high is the temperature?
- Has she tried antipyretics?
- When the patient wakes up, is she playful/acting at her baseline? What is her activity like right now?
- Is she drinking fluids?
- How many wet diapers has she had today?
- Any additional symptoms? (Cough, congestion, rhinorrhea, difficulty breathing, vomiting, diarrhea, rash)
- Has she been vaccinated?
- Severity/Management:
  - Emergency room if concern for lethargy and/or dehydration
  - Urgent next day appointment if seems tired but not lethargic and well-hydrated
  - Can consider supportive care/reassurance if solely URI symptoms, particularly in an older child, who is behaving normally, well-hydrated, and afebrile

1. **A parent calls to say her 6 year old with asthma has been wheezing all night and wants you to prescribe medicine.**
   - What is the patient’s asthma history? Any history of hospitalizations, particularly to the ICU requiring positive pressure or intubation?
   - What are the patient’s current medications? (Can try to assess intermittent vs. persistent asthma based on use of controller meds)
   - Have they tried albuterol or any other medications today? When was the last treatment and did it help?
   - What other symptoms is he experiencing? Fevers? URI symptoms? Allergy symptoms?
   - Can the parent provide an objective assessment of his respiratory status? (Ask them about his general appearance, describe breathing pattern, count a respiratory rate)
   - Severity/Management
     - Emergency room if requiring albuterol more than every 4 hours without improvement, lethargy, respiratory distress, cyanosis
     - Urgent next day appointment if albuterol is helping and patient is breathing comfortably – ensure no red flags, i.e. prior intubation, very severe or poorly controlled asthma
     - If has not trialed any medications, you may recommend trialing albuterol treatment. If they do not have at home, review your practice’s over-the-phone medication prescription policy including renewals and new medications.
2. **A parent calls after her 4 month old rolled off the bed and fell. She is very nervous and wants to know what to do?**
   - How long ago did the child fall?
   - How high is the bed?
   - Did he hit his head?
   - Did he lose consciousness?
   - Is he vomiting?
   - Is he acting normally or more tired than usual?
   - Did he injure any other part of his body?
   - Any notable physical exam findings? (bumps on the head, lacerations or bruising, change in mental status, current activity level)
   - Severity/Management
     - Emergency room if any concerning history – lethargy, vomiting, change in behavior, palpable skull fracture, lacerations, loss of consciousness, or severe mechanism of injury (i.e. fall from >3 ft, head struck at high impact)
       - - Review the PECARN guidelines^1^ for head trauma
     - Urgent next day appointment if very minor fall, behaving completely normally, no loss of consciousness, and no other concerns
3. **A parent calls that her 1 month old hasn’t stooled in 3 days and is crying a lot. She wants to know what she can give him to help with his constipation.**
   - Does the child have any significant birth history?
   - Do they have any prior medical problems or hospitalizations?
   - What does he normally eat? Have there been any changes with his feeding patterns?
   - What were his stools like previously? Soft or hard? Did he pass meconium right after birth?
   - Has he had any vomiting? If so, projectile vs. spit up?
   - What is his activity level? Is he behaving normally?
   - Do the parents think his belly looks more distended? Does it seem painful to touch?
   - Severity/Management
     - If no red flags based on above questioning, can likely provide reassurance over the phone, thus preventing an office or ED visit. Discuss indications for call-back, urgent appointment, or any symptoms that would necessitate an ED visit.

Reference:

1. Kuppermann N, Holmes J, Dayan P, Hoyle J. Identification of children at very low risk of clinically important brain injuries after head trauma: a prospective cohort study. Lancet. 2009.
