# Supplementary material for: A Curriculum to Improve Pediatric Residents' Telephone Triage Skills
Source: MedEdPORTAL. 2020 Oct 22;16:10993. doi: 10.15766/mep_2374-8265.10993 (PMC7586755; doi:10.15766/mep_2374-8265.10993)
Supplement: Supplementary file 1 — Pediatric Phone Triage Conference Presentation.pptxFaculty Guide - Pediatric Phone Triage Conference.docxJust-in-Time Training.docxResident Cheat Sheet.docxPre- and Postexperience Self-Assessment.docxConvenience Sample Preassessment.docx [file mep_2374-8265.10993-s001.zip › D. Resident Cheat Sheet.docx]

Telephone Triage “Cheat-Sheet”

**Helpful Resources:**

Poison Control: 1-800-222-1222 Practice Guidelines: AAP.org

Medication Dosing: Lexicomp.com General Info: Uptodate.com
