# Supplementary material for: A Curriculum to Improve Pediatric Residents' Telephone Triage Skills
Source: MedEdPORTAL. 2020 Oct 22;16:10993. doi: 10.15766/mep_2374-8265.10993 (PMC7586755; doi:10.15766/mep_2374-8265.10993)
Supplement: Supplementary file 1 — Pediatric Phone Triage Conference Presentation.pptxFaculty Guide - Pediatric Phone Triage Conference.docxJust-in-Time Training.docxResident Cheat Sheet.docxPre- and Postexperience Self-Assessment.docxConvenience Sample Preassessment.docx [file mep_2374-8265.10993-s001.zip › E. Pre- and Postexperience Self-Assessment.docx]

Appendix E: Pre- and Postexperience Self-Assessment

Regarding your experience **BEFORE** taking ambulatory call:

- **How well could you take a history over the phone from a parent?**

[] Could do very well [] Could do well [] Not sure [] Could not do well [] Could not do at all

- **How well could you appropriately triage a patient over the phone?**

[] Could do very well [] Could do well [] Not sure [] Could not do well [] Could not do at all

Regarding your experience **AFTER** taking ambulatory call:

- **How well can you take a history over the phone from a parent?**

[] Can do very well [] Can do well [] Not sure [] Can’t do well [] Can’t do at all

- **How well can you appropriately triage a patient over the phone?**

[] Can do very well [] Can do well [] Not sure [] Can’t do well [] Can’t do at all

Logistics:

- **Did you have a one on one review session prior to taking ambulatory call?**
  1. Yes
  2. No
- **If you DID have a session, how well did it prepare you for your calls?**
  1. Well prepared
  2. Prepared
  3. Not sure
  4. Poorly prepared
  5. Not prepared at all
- **If you DID NOT have a session, do you feel like you needed one?**
  1. Yes
  2. No
  3. Neutral
- **Would you take ambulatory call if offered again?**
  1. Yes
  2. No
  3. Neutral
- **What year are you?**
  1. PGY2
  2. PGY3

Comments? ___________________________________________________________________________________ _____________________________________________________________________________________________
