# Supplementary material for: A Curriculum to Improve Pediatric Residents' Telephone Triage Skills
Source: MedEdPORTAL. 2020 Oct 22;16:10993. doi: 10.15766/mep_2374-8265.10993 (PMC7586755; doi:10.15766/mep_2374-8265.10993)
Supplement: Supplementary file 1 — Pediatric Phone Triage Conference Presentation.pptxFaculty Guide - Pediatric Phone Triage Conference.docxJust-in-Time Training.docxResident Cheat Sheet.docxPre- and Postexperience Self-Assessment.docxConvenience Sample Preassessment.docx [file mep_2374-8265.10993-s001.zip › F. Convenience Sample Preassessment.docx]

Appendix F: Convenience Sample Self-Assessment

(Survey for residents who did NOT participate in the curriculum)

1. H**ow well can you take a history over the phone from a parent?**
   1. Can do very well
   2. Can do well
   3. Not sure
   4. Can’t do well
   5. Can’t do at all
2. **How well can you appropriately triage a patient over the phone?**
   1. Can do very well
   2. Can do well
   3. Not sure
   4. Can’t do well
   5. Can’t do at all
3. **What year are you?**
   1. PGY1
   2. PGY2
   3. PGY3
4. **What clinic are you in?**
   1. Washington Heights
   2. Broadway
   3. Audubon
   4. Rangel
