# Supplementary material for: A Curriculum to Improve Pediatric Residents' Telephone Triage Skills
Source: MedEdPORTAL. 2020 Oct 22;16:10993. doi: 10.15766/mep_2374-8265.10993 (PMC7586755; doi:10.15766/mep_2374-8265.10993)
Supplement: Supplementary file 1 — Pediatric Phone Triage Conference Presentation.pptxFaculty Guide - Pediatric Phone Triage Conference.docxJust-in-Time Training.docxResident Cheat Sheet.docxPre- and Postexperience Self-Assessment.docxConvenience Sample Preassessment.docx [file mep_2374-8265.10993-s001.zip › _Educational_Summary_Report_10993.pdf]

MedEdPORTAL® | The AAMC Journal of Teaching and Learning Resources

Original Publication Open Access

A Curriculum to Improve Pediatric Residents' Telephone Triage Skills

Lauren T. Roth, MD*, Mariellen Lane, MD, Suzanne Friedman, MD

*Corresponding Author: laroth@montefiore.org

Abstract

Introduction: Telephone triage systems are frequently used due to their success in decreasing emergency department utilization, reduction of health care costs, and high levels of satisfaction among patients and providers. Despite phone triage's prevalence, few residency programs have designated curricula for residents to learn this vital skill. **Methods:** We designed a phone triage curriculum initially piloted with senior residents at one of our continuity clinics. The curriculum consisted of a didactic session, a just-in-time simulation training session, and an experiential component of being on call during the ambulatory rotation. Retrospective pre-post self-assessments evaluated resident perceptions of their skills in taking histories and triaging care over the phone in addition to obtaining qualitative feedback from faculty and residents immediately after the curriculum and 1-2 years postgraduation. **Results:** Of 11 eligible residents, 10 (91%) chose to participate in the pilot curriculum. Residents reported that their skills in history taking over the phone improved from 20% to 90% and their ability to triage patients over the phone improved from 0% to 80%. This led to a quality improvement initiative to increase patient calls and has continued for 5 years, with continued positive feedback from residents and attendings. **Discussion:** Phone triage skills are a necessity for pediatric providers, but few residency programs have training curricula in place. Through an experience-based phone triage program, residents significantly improved their self-reported skills at history taking and triaging. Similar curricula could easily be adopted at other institutions.

Keywords:
Phone Triage, Resident Education, Pediatrics, Case-Based Learning, Clinical/Procedural Skills Training

Educational Objectives

At the completion of this curriculum, learners will be able to:

1. Take a complete and appropriate patient history with all essential clinical information from a family member over the phone.
2. Effectively triage patients' clinical needs based on common presentations and red flags in the history they have taken over the phone.
3. Improve confidence in autonomous decision-making skills while performing telephone triage without direct supervision.

Introduction

Due to a worldwide pandemic and shifting of medical resources, telephone triage systems are being utilized more than ever before. Telephone triage systems are designed for patients and families to call and have their concerns addressed by a medical provider in an effort to determine whether they require emergent or urgent medical care.[1] They are critical for risk management, are the cornerstone of demand-management, and provide patients with information to assist with decision-making regarding how to appropriately utilize health care services.[1]

Both physicians and patients have reported high levels of satisfaction with telephone triage services.[2-4] By allowing families to call ahead for triage, providers can often eliminate the need for patients to attend in-person sick visits, which can decrease the need for patients and families to miss work or school. Telephone triage services have been associated with reductions in emergency department utilization, particularly when available after hours.[3,5,6] One study specifically showed decreased utilization of the emergency room by uninsured and Medicaid patients, which can improve emergency room crowding and significantly decrease health care costs.[6] Referrals to the emergency room from telephone triage have been shown to be more appropriate than referrals from elsewhere,[5] and the vast majority of families follow the advice provided, with

Citation:
Roth LT, Lane M, Friedman S. A curriculum to improve pediatric residents' telephone triage skills. *MedEdPORTAL*. 2020;16:10993.
https://doi.org/10.15766/mep_2374-8265.10993

Copyright © 2020 Roth et al. This is an open-access publication distributed under the terms of the Creative Commons Attribution license.

compliance rates ranging from 74% to 94% for recommendations to be evaluated urgently or to be treated at home.[5,7,8] Patients often receive medication refills and referrals for specialty care through telephone triage, eliminating unnecessary in-person visits.[2] Thus, telephone triage systems have been shown to be a safe and efficient gatekeeper for health care resources.

For many of these reasons, telephone triage systems continue to increase in prevalence. In 2000, an estimated 35 million U.S. citizens accessed telephone triage services, with an expected 25% increase per year.[1] Telephone triage accounts for nearly 25% of patient encounters in internal medicine[1] and approximately 27% in pediatrics.[9] The American Academy of Pediatrics reports that as of 2012, 56% of pediatricians responded to all after-hours calls, 30% utilized a call center with an on-call physician, and 20% had registered nurses perform the initial triage with physician support as needed.[10] Even during office hours, office staff or nurses may answer calls, but the majority of pediatricians respond to these messages to triage patients over the telephone themselves.[10]

Despite the widespread use of telephone triage by physicians, very few training programs have telephone triage curricula in place.[9,11-13] Only 6% of residency programs in the U.S. provide training in telephone triage.[11] In a survey of internal medicine program directors, the majority felt residents were not prepared to perform phone triage, with under 10% of faculty reporting residents being adequately prepared for these calls.[11] In pediatrics, fewer than half of residency programs offer specific training in telephone triage.[9,13] Residents have repeatedly cited telephone triage as a significant gap in their training and feel minimally prepared to manage problems by telephone after graduation.[14-16]

Since the majority of physicians are required to provide some form of telephone triage, training them for this practice is vital for its success. In the few studies that have been published, specific training in telephone triage has been shown to improve history taking[12] and patient management over the phone.[17,18] Still, despite significant advances in technology and shifting practices to include more telephone triage, there have been no new published training materials since the early 2000s. Given this significant gap in the literature and a perceived gap in knowledge at our own institution, we developed a curriculum to help residents develop phone triage skills and experience taking after-hours phone calls at one of our pediatric continuity clinics.

Methods

Context

Within our urban academic tertiary care hospital, residents see patients in one of four continuity clinics. At all sites, after-hours calls are covered by the attending physician faculty, who are the first line of triage. As part of the mandatory residency curriculum, all residents are required to complete a 1-month ambulatory rotation each postgraduate year. The telephone triage curriculum was offered to residents during this scheduled 1-month rotation at their designated continuity clinic. The curriculum was initially optional and piloted with second- and third-year residents at one of the four practice sites in our ambulatory care network. At this site, there are eight attending physicians and 19 residents. Residents who opted into the curriculum were assigned to be on call 1 night per week over the course of their 4-week rotation, thereby having the opportunity to have 4 nights of practice performing phone triage. Each resident was assigned a faculty member as backup to assist with any questions that arose and to provide feedback at the end of each session. Learners were expected to have some general pediatrics knowledge but no prior experience performing phone triage.

Curriculum Development

The curriculum was designed utilizing Kern's six-step approach to curriculum development for medical education.[19] A general needs assessment was based on a literature review, which showed a significant gap in current evidence and material on this topic. At our institution, a targeted needs assessment was based on faculty perceptions and resident desire. All attending physicians surveyed were interested in having residents take call with them and felt that training was necessary for residents to perform telephone triage. We developed goals and objectives based on provider feedback and ensured they upheld the requirements outlined by the Accreditation Council for Graduate Medical Education for continuity clinic experience. We reviewed various educational strategies and designed the curriculum to be implemented in three parts: a didactic educational session, a secondary one-on-one training prior to initiating call, and the experiential component of being on call for 4 nights during the rotation. Given scheduling restraints, we knew that a single lecture for all residents likely would not suffice since each resident had ambulatory rotation at different times during the year. Given these scheduling restraints, we decided to combine the didactic session with just-in-time training, which has been shown to be an effective tool for improving clinical skills and is perceived favorably by residents.[20-22] Prior studies have shown that simulation and role-play in telephone triage improve resident

Copyright © 2020 Roth et al. This is an open-access publication distributed under the terms of the Creative Commons Attribution license.

history taking and overall management,[12,17,18] so we aimed to develop a just-in-time training simulation prior to residents taking call. We then piloted the curriculum with a small subset of residents and revised it based on quantitative and qualitative evaluations.

Curriculum Design

The didactic session was a 1-hour conference for all residents scheduled during their daily educational lecture time. This conference aimed to disseminate telephone triage skills facilitated by a PowerPoint presentation and cases to review common scenarios (Appendix A). It was designed to be interactive and appropriate for medical students and residents. An element of role-play was used for the various cases even in this large-group setting. Either individual residents alternated role-playing the on-call doctor or multiple residents played the same role at once. Thus, the conference could be done with both small and large groups. Our didactic session was led by a continuity clinic preceptor with experience in telephone triage who utilized a detailed step-by-step facilitator guide (Appendix B). Using this guide, the conference could be led by any physician with some knowledge of general pediatrics and experience in telephone triage.

A second one-on-one just-in-time training was then given to select residents prior to them starting to take call (Appendix C). Before their first night of call, residents met one-on-one with one of the faculty members to receive an introduction to phone triage, discuss the logistics of taking call, and review triaging techniques. This meeting included common types of calls, specific skills and techniques for accurately assessing the patient over the phone, and discussion of the triage process. The training was led by the faculty member as a question-and-answer session to ensure the resident understood the basic process and how best to assess the history and physical exam findings over the phone. Additionally, the faculty member reviewed multiple practice cases via simulation of a call to ensure the resident felt comfortable and would be prepared for a variety of scenarios. The cases simulated were based on prior studies outlining the most common chief complaints from patient calls.[18,23] The residents were given a copy of this information for reference in addition to a one-page cheat sheet for quick reminders (Appendix D).

The experiential component allowed residents to be on call 1 night per week during the course of the 4-week ambulatory rotation. This entailed providing the clinic's telephone triage system with the resident's direct phone number so the resident could be easily accessed between the hours of 5:00 pm and 9:00 am. Any patient who called during those hours was forwarded to the resident on call. Residents were expected to answer any clinical questions and to triage the patients' needs. Depending on the complexity of a patient's issue, residents could engage with the assigned faculty member after each call or summarize the next morning to ensure their responses were appropriate and to ask any questions that may have arisen. The following day, residents had the opportunity to debrief all of their calls with a faculty member to ensure maximum skill development.

Evaluation

To evaluate the curriculum, we utilized retrospective pre- and postsurveys, which are particularly useful when developing a brand-new curriculum because learners often "don't know what they don't know" and can end up using different internal standards when completing evaluations in the traditional pre- and postexperience manner.[24-26] They are also a good method of evaluation to prevent compromising anonymity when there is a small sample size.[24] Residents who participated in the curriculum completed a retrospective pre- and postexperience self-assessment of skills and perceptions of the program with a qualitative free-text assessment (Appendix E). Utilizing a 5-point Likert scale, they were asked to evaluate how well they were able to take a history over the phone from a parent both before and after taking ambulatory call, in addition to how well they could appropriately triage a patient over the phone both before and after taking call. They were also asked how well the sessions prepared them for their call and whether they would take ambulatory call if offered again. Finally, there was a free-text assessment for any additional comments.

The same self-assessment was administered to a convenience sample of residents who received only the didactic portion of the curriculum and did not receive the one-on-one training or participate in ambulatory call (Appendix F). These responses were compared with the select residents who had completed the full curriculum and engaged in the after-hours call system to ensure the responses were similar to the study group's retrospective responses, thus eliminating the possibility of recall bias (since the assessments were retrospective) or selection bias (since the study was optional).

Lastly, we surveyed all general pediatricians and fellows in pediatric specialties who participated in later iterations of this experience for qualitative feedback and relevance to their current practice. Preceptors who participated in the phone triage program were also surveyed regarding their experience and perceived skills of the residents.

Copyright © 2020 Roth et al. This is an open-access publication distributed under the terms of the Creative Commons Attribution license.

Results

Eleven residents were eligible to participate in this optional curriculum. They were second- or third-year pediatric residents who did their ambulatory rotation at the Washington Heights Family Health Center Pediatric Practice. Of the 11 eligible residents, 10 (91%) chose to participate in the telephone triage curriculum. One hundred percent of these residents completed the end-of-curriculum assessment. Six were PGY 2, and four were PGY 3. Of these residents, 30% went on to careers in general pediatrics.

Baseline responses from the residents who completed the curriculum ($n = 10$) were compared with a convenience sample of residents ($n = 8$) who participated in only the didactic portion to ensure there was no selection bias when compared to the residents who opted into the curriculum. This was also done to assess for recall bias in those who completed the curriculum. At baseline, only 20% of residents who completed the curriculum and 25% of the convenience sample reported that they could take a history over the phone well or very well ($p = 1.00$). Additionally, at baseline, 0% of residents who completed the curriculum and 12% of the convenience sample reported that they could triage patients over the phone well or very well ($p = .4737$). Thus, there was no significant difference in the perceived knowledge and skills of residents who did not complete the curriculum or who did but reported their precurricular attitudes retrospectively.

After the full curriculum, 90% reported that they could take a phone history well or very well compared with the baseline 20% ($p < .05$). Additionally, after the curriculum, 80% of residents reported they could triage patients over the phone well or very well compared with the baseline 0% ($p < .05$). Three residents did not have a one-to-one review session immediately prior to taking call due to logistical reasons, and two of the three stated they would have liked one. One hundred percent of residents who had the one-on-one session with a preceptor felt well prepared to perform phone triage. Nine residents (90%) stated they would take ambulatory call again if offered, and one (10%) felt neutral.

Qualitative feedback was elicited from the residents who participated in the pilot curriculum immediately after the experience. Since this curriculum continued for 5 years, residents who participated in later iterations of the curriculum were surveyed 1-2 years after graduation to determine its impact on their careers in both primary and specialty care. Attending physicians who precepted residents in phone triage were also surveyed regarding their interest in the program and their perceived skills of the residents. Common themes included the benefit of the experience, its applicability to a variety of specialties, skill gained, and the need to continue and spread the program (see the Table). As part of the formal ambulatory rotation's evaluation, one resident specifically highlighted the telephone triage system: "Overnight call was a good learning experience." Attending physicians also provided feedback on how to improve the educational experience for the residents, such as by setting clear expectations about when to call the attending, assessing the experience of the resident to determine how much autonomy to provide, evaluating specific concerns and learning interests ahead of time, and increasing the exposure overall.

Almost all residents and faculty members were interested in seeing this curriculum continue and expand. This led to the development of a clinic-wide, resident-led quality improvement project to increase the number of patient calls, not only to improve residency learning through increased experience but also because a gap in patient care was seen. This quality improvement project was a 2-year effort to decrease emergency department utilization by enhancing the phone triage program. The average baseline number of calls was 6.5 per week and increased to 8.5 per week, with a peak of 21 calls throughout this initiative. Telephone triage is now a mandatory part of the curriculum, and over 100 residents have participated.

Discussion

Phone triage skills are a necessity for pediatric providers, but few residency programs have training curricula in place. Through a simulation- and experience-based phone triage curriculum, residents significantly improved their self-reported skills at taking patient histories and triaging patients over the phone. Implementation of this curriculum was met with positivity from both residents and faculty, and postgraduates in both primary care and various specialties have cited the importance of the curriculum in their daily practice and the desire for further training. Faculty members need only to have some experience performing phone triage and general pediatrics knowledge in order to train residents on these necessary skills.

Some of the lessons we learned while developing this curriculum are that not all possible patient concerns could be addressed and practiced prior to residents performing telephone triage. Thus, residents were often required to use prior knowledge, skills, and experience to triage phone calls with the option of calling their attending if they had any questions. Additionally, call volume varies significantly from day to day, so we could not guarantee how many patient calls a resident would answer in a given night.

Copyright © 2020 Roth et al. This is an open-access publication distributed under the terms of the Creative Commons Attribution license.

Table. Thematic Quotes

Theme	Immediately Postcurriculum	1-2 Years Postcurriculum	Attending Preceptors
Positive experience	"Good experience for residents." "Good opportunity and exposure that we don't get elsewhere in residency."	"Being able to take after hours calls as a resident with an attending as backup was an immensely valuable experience." "I really enjoyed the experience because it was a different kind of challenge to assess a situation over the phone with no face to face contact."	"[Residents are] excellent. Do not need to micromanage." "Residents should participate in phone triage. It is an excellent preparation for telemedicine."
Applicability	"I think it would be very valuable for anyone, particularly those going into primary care." "Good experience at something we will have to do regardless of specialty."	"Telephone management and triage of pediatric problems is a large part of my daily practice. It is a challenging skill, but one that I have found to be of utmost importance in bringing children the care they need." "As an attending who has now been on call via telephone many times, I can truly appreciate the extra training as telephone calls involve new skills we are not exposed to during residency."	"It is important for residents to learn. No matter what specialty residents go into they will need to be able to respond to patient phone calls." "I think it is important for residents to gain confidence in phone triage. It will help with primary care but also make them more confident about dealing with issues after hours even if they go into a specialty."
Skills attained	"Helped me learn how to ask questions over the phone to get the information needed to triage." "Forces one to think quickly about disposition/triage without seeing the patient."	"I had the chance to think critically about my comfort level with patients staying home versus seeking further care." "The phone triage curriculum was instrumental in developing my comfort and confidence in being on call… I received calls across a wide range of outpatient pediatric issues… and was able to think about my own management plan in real time." "I learned how to ask for verbal confirmation of specific exam findings without being able to see the patient."	"I think that phone triage is an essential skill for training. It allows the resident to assess symptoms and signs from families and make real time assessments of who needs to be seen, who can be deferred to the next day and give real time advice. These skills also translate into subspecialty care where providers perform phone triage with families/patients." "[Phone triage] is a test of their investigatory and communication skills."
Further spread	"I wish we had more calls so we could practice more." "Please keep [this curriculum]… everyone should have the option."	"There should be more training in phone triage as a medical skill in residency." "Part of my job now is taking calls from EDs across the region for potential transfer, and triaging a patient without seeing them is a totally unique skill that I wish I had done more of in residency"	"It should be part of their rotation in ambulatory" "It would be good to know what residents are concerned about when they take phone calls—what they view as challenging calls and then we can give them feedback or guidance accordingly." "[Residents] would benefit from increased experience."

Abbreviation: ED, emergency department.

This is similar to a resident continuity clinic where the patient show-rate often varies. Given the interest in furthering these skills, we have made significant efforts to increase the number of patient calls. The curriculum would be more robust if residents had multiple opportunities to practice ambulatory call throughout the year, in addition to expanding to include specialties that frequently use telephone triage. Through verbal feedback, we learned that residents found the one-on-one training much more impactful than the larger didactic session since the former was easier to schedule and occurred closer to the experiential component. The didactic portion is now utilized to introduce the curriculum and garner early buy-in from residents. We also learned the importance of preceptors being very clear about their expectations for support (i.e., whether to contact them after every call or at set times in the night, or to debrief in the morning) since preceptors and residents all vary. The simulation is also best done when the facilitator knows the resident well, since the facilitator can more easily assess the learner and tailor the cases to the learner's baseline skill level. As residents gain more experience in telephone triage, the just-in-time training works best when they can build on their prior experiences and refer back to specific calls they have received.

This study was limited by its very small sample size—only 10 residents initially piloted the curriculum. However, given the overwhelmingly positive feedback based on this pilot study, the curriculum was spread to all other continuity clinics within our ambulatory care network and is now a mandatory part of the resident curriculum. We did not continue pre- and postexperience evaluation but instead made changes based on real-time feedback. This curriculum has now been used for 5 years, with 2 of those years being a formal quality improvement project to increase patient calls and decrease emergency room utilization that was very successful. Another limitation to this study is that we did not formally evaluate all of the objectives. We assessed perceived improvement in history taking and triage as opposed to an actual skills assessment, which would have made the data stronger. In reality, when preceptors perform the just-in-time training, they continue to go through cases and debrief any incorrect management plans until the resident has a clear understanding of the appropriate plan, although this is not formally evaluated. Future efforts could focus on performing true skills assessments through either simulation or faculty observation. Finally, since our pilot curriculum was optional, the residents who chose to participate may pose a

Copyright © 2020 Roth et al. This is an open-access publication distributed under the terms of the Creative Commons Attribution license.

self-selection bias in that they may have been more interested and motivated in learning the skills necessary to triage patients over the telephone. However, after spreading this curriculum to all continuity clinics, nearly all residents have stated they were interested and motivated in improving their phone triage skills.

Telephone triage has been used in medical practices for decades, but it is now rapidly expanding in the COVID-19 pandemic due to decreasing face-to-face visits to minimize exposure, as well as public fear of viral spread. Telephone triage systems allow providers to offer reassurance and answer questions while patients and families remain safely at home. More importantly, providers can assess whether there is a true emergency and emphasize the importance of seeking care during a time when families may fear to utilize the medical system. Full virtual appointments are now becoming readily available, but in order to effectively practice telemedicine, providers must be able to triage which patients are eligible for a virtual as opposed an to in-person visit. As technology advances and telemedicine becomes more widespread, training physicians for telephone triage is vital for its success.

We believe this curriculum can be universally applied regardless of specialty. Almost every specialty uses telephone triage in some capacity. Many specialties utilize triage similarly to primary care in that patients can call directly[27-29]; some use phone triage when speaking with other health care providers in efforts to determine patient acuity when being referred to the emergency room or need to transfer between hospitals. While this practice may be different from speaking to patients, learning the skill of triaging without performing an in-person history and physical exam is still vital to its success. Even in clinics that utilize a call center or nurse as the first line of triage, a physician almost always plays some role in the triage process, often as the final voice. In qualitative feedback, fellows in various specialties who completed this curriculum all expressed gratitude for the experience, and many stated they would have liked more experience prior to fellowship and believe we should continue and broaden this program for future residents.

Overall, this curriculum showed a significant improvement in resident-reported skills in taking patient histories and triaging over the phone. We believe this curriculum is very important for resident education of phone triage skills and could be easily adopted by other institutions. Most pediatric practices already have a phone triage system in place, so adapting it to involve residents is likely a broadly achievable goal. As a result of this pilot curriculum, residents from all clinic sites in our ambulatory care network are now required to participate in the program and continue to express interest and positive outcomes from their training experience. As technology advances and health care utilization continues to shift, training physicians to perform this vital skill should be a priority across residency programs.

Appendices

A. Pediatric Phone Triage Conference Presentation.pptx
B. Faculty Guide - Pediatric Phone Triage Conference.docx
C. Just-in-Time Training.docx
D. Resident Cheat Sheet.docx
E. Pre- and Postexperience Self-Assessment.docx
F. Convenience Sample Preassessment.docx

All appendices are peer reviewed as integral parts of the Original Publication.

Lauren T. Roth, MD: Instructor, Department of Pediatrics, Montefiore Medical Center; ORCID: https://orcid.org/0000-0001-5592-3927

Mariellen Lane, MD: Associate Professor, Department of Pediatrics, Columbia University Irving Medical Center

Suzanne Friedman, MD: Assistant Professor, Department of Pediatrics, Columbia University Irving Medical Center

Disclosures
None to report.

Funding/Support
None to report.

Ethical Approval
Reported as not applicable.

References

1. Coons KC, DuMoulin JP. *Telephone Triage: White Paper of the American College of Physicians–American Society of Internal Medicine*. American College of Physicians; 2000. https://www.acponline.org/acp_policy/policies/telephone_triage_diagnostic_techniques_procedures_2000.pdf
2. Caralis P. Teaching residents to communicate: the use of a telephone triage system in an academic ambulatory clinic. *Patient Educ Couns*. 2010;80(3):351-353. https://doi.org/10.1016/j.pec.2010.07.028
3. O'Connell JM, Johnson DA, Stallmeyer J, Cokingtin D. A satisfaction and return-on-investment study of a nurse triage service. *Am J Manag Care*. 2001;7(2):159-169.

Copyright © 2020 Roth et al. This is an open-access publication distributed under the terms of the Creative Commons Attribution license.

4. Poole SR, Schmitt BD, Carruth T, Peterson-Smith A, Slusarski M. After-hours telephone coverage: the application of an area-wide telephone triage and advice system for pediatric practices. *Pediatrics*. 1993;92(5):670-679.
5. Barber JW, King WD, Monroe KW, Nichols MH. Evaluation of emergency department referrals by telephone triage. *Pediatrics*. 2000;105(4):819-821. https://doi.org/10.1542/peds.105.4.819
6. Howell T. ED utilization by uninsured and Medicaid patients after availability of telephone triage. *J Emerg Nurs*. 2016;42(2): 120-124. https://doi.org/10.1016/j.jen.2015.08.015
7. Crane JD, Benjamin JT. Pediatric residents' telephone triage experience: do parents really follow telephone advice? *Arch Pediatr Adolesc Med*. 2000;154(1):71-74.
8. Kempe A, Bunik M, Ellis J, et al. How safe is triage by an after-hours telephone call center? *Pediatrics*. 2006;118(2): 457-463. https://doi.org/10.1542/peds.2005-3073
9. Wood PR. Pediatric resident training in telephone management: a survey of training programs in the United States. *Pediatrics*. 1986;77(6):882-885.
10. Periodic Survey #79 Telephone Care. American Academy of Pediatrics. 2012. https://www.aap.org/en-us/professional-resources/Research/pediatrician-surveys/Pages/Periodic-Survey-79-Telephone-Care.aspx
11. Flannery MT, Moses GA, Cykert S, et al. Telephone management training in internal medicine residencies: a national survey of program directors. *Acad Med*. 1995;70(12):1138-1141.
12. Wood PR, Littlefield JH, Foulds DM. Telephone management curriculum for pediatric interns: a controlled trial. *Pediatrics*. 1989;83(6):925-930.
13. Barratt MS, Tanz RR. A survey of the structure and function of pediatric continuity clinics. *Am J Dis Child*. 1992;146(8):937-940. https://doi.org/10.1001/archpedi.1992.02160200059027
14. Roberts KB, Starr S, DeWitt TG. The University of Massachusetts Medical Center office-based continuity experience: are we preparing pediatrics residents for primary care practice? *Pediatrics*. 1997;100(4):e2. https://doi.org/10.1542/peds.100.4.e2
15. Rosenberg AA, Kamin C, Glicken AD, Jr Jones MD. Training gaps for pediatric residents planning a career in primary care: a qualitative and quantitative study. *J Grad Med Educ*. 2011;3(3):309-314. https://doi.org/10.4300/jgme-d-10-00151.1
16. Dumont-Driscoll MC, Barbian LT, Pollock BH. Pediatric residents' continuity clinics: how are we really doing? *Pediatrics*. 1995;96(4):616-621.
17. Ottolini MC, Greenberg L. Development and evaluation of a CD-ROM computer program to teach residents telephone management. *Pediatrics*. 1998;101(3):e2. https://doi.org/10.1542/peds.101.3.e2
18. Benjamin JT. Pediatric residents' telephone triage experience: relevant to general pediatric practice? *Arch Pediatr Adolesc Med*. 1997;151(12):1254-1257. https://doi.org/10.1001/archpedi.1997.02170490080014
19. Thomas PA, Kern DE, Hughes MT, Chen BY, eds. *Curriculum Development for Medical Education: A Six-Step Approach*. 3rd ed. Johns Hopkins University Press; 2016.
20. Mangum R, Lazar J, Rose MJ, Mahan JD, Reed S. Exploring the value of just-in-time teaching as a supplemental tool to traditional resident education on a busy inpatient pediatrics rotation. *Acad Pediatr*. 2017;17(6):589-592. https://doi.org/10.1016/j.acap.2017.04.021
21. Schuller MC, DaRosa DA, Crandall ML. Using just-in-time teaching and peer instruction in a residency program's core curriculum: enhancing satisfaction, engagement, and retention. *Acad Med*. 2015;90(3):384-391. https://doi.org/10.1097/acm.0000000000000578
22. Kamdar G, Kessler DO, Tilt L, et al. Qualitative evaluation of just-in-time simulation-based learning: the learners' perspective. *Simul Healthc*. 2013;8(1):43-48. https://doi.org/10.1097/sih.0b013e31827861e8
23. Belman S, Chandramouli V, Schmitt BD, Poole SR, Hegarty T, Kempe A. An assessment of pediatric after-hours telephone care: a 1-year experience. *Arch Pediatr Adolesc Med*. 2005;159(2):145-149. https://doi.org/10.1001/archpedi.159.2.145
24. Lang D, Savageau J. Starting at the end: measuring learning using retrospective pre-post evaluations. *AEA365 blog*. July 31, 2017. https://aea365.org/blog/starting-at-the-end-measuring-learning-using-retrospective-pre-post-evaluations-by-debi-lang-and-judy-savageau/
25. Bhanji F, Gottesman R, de Grave W, Steinert Y, Winer LR. The retrospective pre–post: a practical method to evaluate learning from an educational program. *Acad Emerg Med*. 2012;19(2): 189-194. https://doi.org/10.1111/j.1553-2712.2011.01270.x
26. Howard GS. Response-shift bias: a problem in evaluating interventions with pre/post self-reports. *Eval Rev*. 1980; 4(1):93-106. https://doi.org/10.1177%2F0193841X8000400105
27. Black KL, Caufield C. Standardization of telephone triage in pediatric oncology. *J Pediatr Oncol Nurs*. 2007;24(4):190-199. https://doi.org/10.1177/1043454207299407
28. Fishe JN, Luberti AA, Master CL, et al. After-hours call center triage of pediatric head injury: outcomes after a concussion initiative. *Pediatr Emerg Care*. 2016;32(3):149-153. https://doi.org/10.1097/pec.0000000000000724
29. Lehmann AE, Kozin ED, Sethi RKV, et al. Resident responses to after-hours otolaryngology patient phone calls: an overlooked aspect of residency training? *Laryngoscope*. 2018;128(5):E163-E170. https://doi.org/10.1002/lary.26784

Received: December 12, 2019
Accepted: June 2, 2020
Published: October 22, 2020

Copyright © 2020 Roth et al. This is an open-access publication distributed under the terms of the Creative Commons Attribution license.
